# Linear ubiquitination prevents lipodystrophy and obesity-associated metabolic syndrome

**DOI:** 10.1126/sciadv.adw2539

**Published:** 2025-09-17

**Authors:** Ximena Hildebrandt, Önay Veli, Armel Hyoubi, Julia Zinngrebe, Ali T. Abdallah, Julian Rodefeld, Anne Hoffmann, Liane Gardeweg, Öykü Kaya, Elena Wagner, Andreas Lindhorst, Matea Poggenberg, Yuan Wang, Joëlle Dimmler, Jutta Schillings, Pegi Koci, Francesca Bonechi, Lucas Valdez Capuccino, Christine Kiefer, Konstantinos Kelepouras, Adhideb Ghosh, Falko Noé, Christian Wolfrum, Michael Singer, Gianmaria Liccardi, Tom Luedde, Aslihan Yavas, Ahmed Ghallab, Jan G. Hengstler, Philipp Antczak, Martin Gericke, Holger Winkels, Matthias Blüher, Henning Walczak, Alessandro Annibaldi, Pamela Fischer-Posovszky, Nieves Peltzer

**Affiliations:** ^1^ Cologne Excellence Cluster on Cellular Stress Responses in Aging-Associated Diseases (CECAD), Cologne, Germany.; ^2^ Centre for Molecular Medicine Cologne (CMMC), University of Cologne, Cologne, Germany.; ^3^ Department of Translational Genomics, Faculty of Medicine, University of Cologne, Cologne, Germany.; ^4^ Department of Genome Editing, Institute of Biomedical Genetics (IBMG), University of Stuttgart, Stuttgart, Germany.; ^5^ Department of Pediatrics and Adolescent Medicine, Ulm University Medical Center, Ulm, Germany.; ^6^ Bioinformatics Core Facility, CECAD Research Center, Cologne, Germany.; ^7^ Medical Department III–Endocrinology, Nephrology, Rheumatology, University of Leipzig Medical Center, Leipzig 04103, Germany.; ^8^ Helmholtz Institute for Metabolic, Obesity and Vascular Research (HI-MAG), Helmholtz Zentrum München at the University of Leipzig and University Hospital Leipzig, Leipzig, Germany.; ^9^ Department of Cardiology, Clinic III for Internal Medicine, University of Cologne, Faculty of Medicine and University Hospital Cologne, Cologne, Germany.; ^10^ Institute of Anatomie, Universität Leipzig, Leipzig, Germany.; ^11^ Genome Instability, Inflammation and Cell death Laboratory, Institute of Biochemistry I, Medical Faculty, University of Cologne, 50931 Cologne, Germany.; ^12^ Institute of Food, Nutrition and Health, ETH, Zurich 8092 Schwerzenbach, Switzerland.; ^13^ Klinik für Gastroenterologie, Hepatologie und Infektiologie, Düsseldorf, Germany.; ^14^ Institut für Pathologie, Universitätsklinikum Düsseldorf, Düsseldorf, Germany.; ^15^ Department of Toxicology, Leibniz Research Centre for Working Environment and Human Factors, Technical University Dortmund, Ardeystr. 67, 44139, Dortmund, Germany.; ^16^ Forensic Medicine and Toxicology Department, Faculty of Veterinary Medicine, South Valley University, Qena, Egypt.; ^17^ Department II of Internal Medicine, Faculty of Medicine and University Hospital Cologne, Cologne, Germany.; ^18^ Institute of Biochemistry I, Medical Faculty, University of Cologne, 50931 Cologne, Germany.; ^19^ German Center for Child and Adolescent Health (DZKJ), partner site Ulm, Ulm, Germany.

## Abstract

Adipocyte hypertrophy during obesity triggers chronic inflammation, leading to metabolic disorders. However, the role of adipocyte-specific inflammatory signaling in metabolic syndrome remains unclear. The linear ubiquitin chain assembly complex, LUBAC, is an E3-ligase that generates nondegradative linear ubiquitination (Lin-Ub). LUBAC regulates NF-κB/MAPK-driven inflammation and prevents cell death triggered by immune receptors like TNF receptor-1. Here, we show that mice lacking HOIP, the Lin-E3 ligase catalytic subunit of LUBAC, in adipocytes (*Hoip^A-KO^*) display lipodystrophy and heightened susceptibility to obesity-induced metabolic syndrome, particularly metabolic dysfunction-associated steatotic liver disease (MASLD). Mechanistically, loss of HOIP attenuates TNF-induced NF-κB activation and promotes cell death in human adipocytes. Inhibiting caspase-8–mediated cell death is sufficient to prevent lipodystrophy and MASLD in *Hoip^A-KO^* obese mice. HOIP expression in adipose tissue positively correlates with metabolic fitness in obese individuals. Overall, our findings reveal a fundamental developmental role for Lin-Ub in adipocytes by mitigating cell death–driven adipose tissue inflammation and protecting against obesity-related metabolic syndrome.

## Teaser

LUBAC preserves adipose tissue integrity via cell death inhibition, preventing metabolic disorders during aging and obesity.

## INTRODUCTION

The pathologic expansion of adipocytes during obesity leads to inflammation within white adipose tissue (WAT). This persistent, low-grade inflammation drives the development of multiple obesity-related metabolic disorders (*1*). While adipocytes are best known for their role in storing lipids, they also have important endocrine functions. They produce a range of signaling molecules, including cytokines such as tumor necrosis factor (TNF) and interleukin-6 (IL-6), and the bona fide adipokines leptin and adiponectin, which modulate inflammatory responses and metabolic processes (*2*). Adipocyte hypertrophy compromises the function and integrity of the whole adipose tissue (AT), contributing to the progression of metabolic diseases (*3*) such as diabetes (*4*), cardiovascular disease (*5*), and metabolic dysfunction-associated steatotic liver disease (MASLD) (*6*). Despite the substantial clinical importance, the precise mechanisms governing the inflammatory role of adipocytes and how these processes exacerbate metabolic complications in obesity remain unclear.

Cytokines of the TNF superfamily (SF) play a crucial role in regulating inflammatory gene activation and cell death responses. Accumulating evidence indicates that TNF-SF members are key drivers of metabolic syndrome (*7*–*10*). While their effect is primarily attributed to inflammatory gene activation via nuclear factor κB (NF-κB) and mitogen-activated protein kinase (MAPK) signaling pathways, the contribution of cell death induced by TNF-SF members is understudied. While it is established that cell death occurs during AT expansion and is linked to metabolic syndrome in obesity (*8*, *11*–*20*), the regulatory mechanisms underlying this process and its specific role in inflammation-related metabolic dysfunction remain poorly understood.

The linear ubiquitin chain assembly complex (LUBAC) is, so far, the only known E3 ubiquitin ligase that generates linear ubiquitin linkage chain types, or linear ubiquitination (Lin-Ub), on target proteins (*21*–*26*). LUBAC consists of HOIL-1 (*Rbck1*), HOIP (*Rnf31*), and SHARPIN (*sipl1*), and it regulates the signaling output of several members of the TNF receptor (TNFR)–SF, with TNFR1 being the most thoroughly characterized. The binding of TNF to its cognate receptor, TNFR1, promotes the assembly of a protein complex that comprises RIPK1, TRAF2, and the E3 ligases cellular inhibitor of apoptosis proteins 1 and 2 (cIAP1/2) and LUBAC, among other proteins (*27*). Ubiquitination events by both cIAP1/2 and LUBAC within this complex promotes the recruitment and activity of TAB1/2/TAK1 and inhibitor of nuclear factor κB kinase α/β (IKKα/β)/Nuclear Factor-kappa B Essential Modulator (NEMO) kinase complexes, resulting in optimal activation of MAPK and NF-κB pathways. Under certain conditions, TNF can promote the assembly of a cell death platform, composed of Fas-associated death domain protein, Caspase-8 and RIPK1 leading to apoptosis. Further binding of RIPK3 to RIPK1 results in activation of the pseudokinase mixed lineage kinase domain-like (MLKL) and execution of necroptosis, an event that is inhibited by caspase-8. Thus, Lin-Ub chains are crucial for optimal NF-κB/MAPK activation and prevention of cell death, making it indispensable for embryogenesis, skin, and immune cell homeostasis and prevention of liver cancer (*21*, *28*–*36*). Patients with inherited mutations in any of these components suffer from several life-threatening immunodeficiencies, auto-inflammation, amylopectinosis (*37*–*40*), and compromised NF-κB responses in nonimmune cells (*41*). A patient carrying a homozygous HOIP loss-of-function variant suffered from hepatomegaly, high blood triglycerides, and wasting. Anti-TNF therapy and low-fat diet temporarily ameliorate some of the clinical symptoms (*40*).

HOIP, the Lin-Ub-E3 ligase component of LUBAC, has been extensively studied in the context of inflammation and immune regulation (*42*), but its role in metabolic health is unknown. In this study, we report that HOIP deficiency in mature adipocytes leads to spontaneous lipodystrophy and increases metabolic syndrome upon diet-induced obesity. Mechanistically, we show that the metabolic disorders, i.e., lipodystrophy and obesity-induced metabolic syndrome, in mice lacking HOIP in adipocytes is driven by caspase-8–mediated cell death. We also provide evidence that Lin-Ub regulates cell death and inflammation in human adipocytes, with LUBAC levels positively correlating with improved metabolic health in obese individuals. This study underscores the role of Lin-Ub in maintaining AT homeostasis and preventing obesity-related metabolic disorders.

## RESULTS

### Lack of linear ubiquitination in adipocytes triggers metabolic syndrome in obese mice

**Fig. 1.**
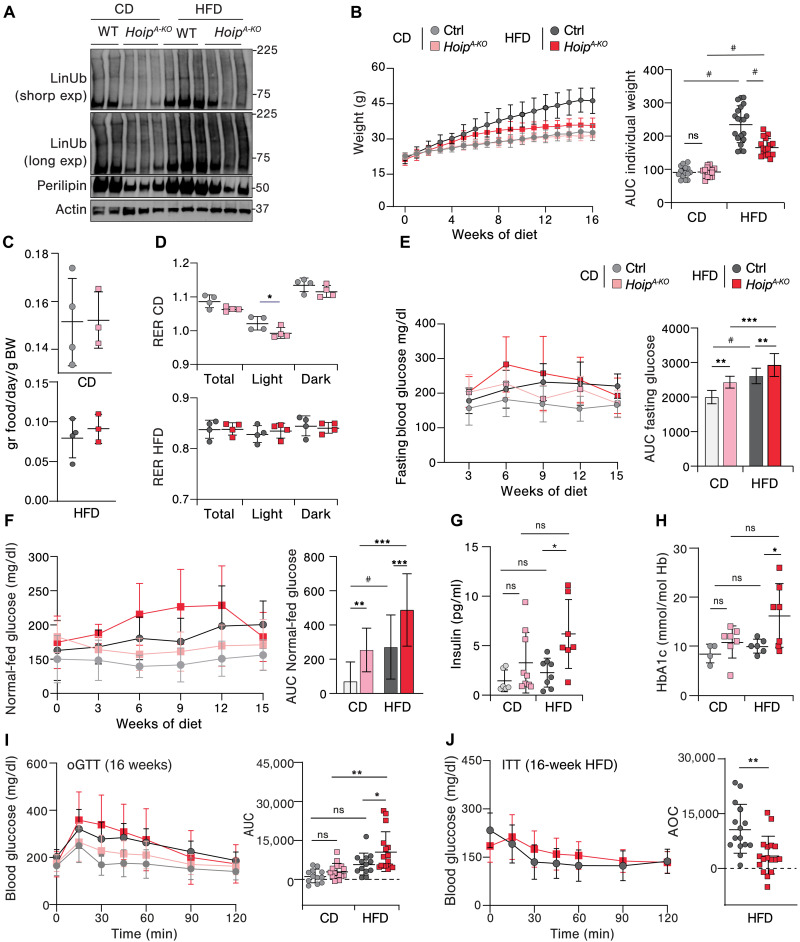
Impaired linear ubiquitination in adipocytes triggers metabolic syndrome in obese mice. (**A**) Western blot of ScWAT protein lysates of WT and *Hoip^A-KO^* mice after 8 weeks of indicated diets and blotted with the indicated antibodies (*n* = 2 to 3). (**B**) Weight monitoring and area under the curve (AUC) of each mouse (*n* = 15 to 20). (**C**) Food consumption (HFD) and (**D**) RER, as monitored using metabolic cages (*n* = 3 to 4). (**E**) Fasting glucose values after 6 hours and mean AUC of the resulting curve (*n* = 9 to 12). (**F**) Normal-fed glucose values and mean AUC of the resulting curve (*n* = 15 to 23). (**G** and **H**) Serum values of insulin (G) and HbA1c (H) at 16 weeks of diet (*n* = 4 to 9). (**I**) oGTT and its individual AUC at 16 weeks of diet (*n* = 12 to 16). (**J**) ITT of HFD and its individual area of curve (AOC) at 16 weeks of indicated diet (*n* = 15 to 17). All values represent the means ± SD. Statistics: [(C), (D), and (J)]: *t* test, *P* values: **P < 0.01 and *P < 0.05; ns, not significant; [(B), (G), (H), and (I)]: One-way ANOVA with selected pair comparison followed by with Bonferroni correction, *P* values GP: #*P* < 0.0001, ***P < 0.0002, **P < 0.0021, and *P < 0.0332; ns, *P* > 0.0333; [(E) and (F)]: AUC of resulting curve followed by two-way ANOVA with Bonferroni correction, *P* values GP: #*P* < 0.0001, ***P < 0.0002, **P < 0.0021, and *P < 0.0332; ns, *P* > 0.0333; *n* of AUC: number of mice. BW, body weight.

To investigate whether Lin-Ub plays a role in obesity-associated metabolic dysfunction, we generated mice lacking HOIP, and therefore Lin-Ub (*33*), in mature adipocytes (fig. S1A) (*Hoip^fl/fl;AdipoqCre^*, hereafter called *Hoip^A-KO^*) and subjected them to a high-fat diet (HFD). Control mice fed with HFD for 16 weeks exhibited an overall increase in Lin-Ub in WAT as compared to mice fed with a control diet (CD) (Fig. 1A), suggesting that LUBAC is activated in obesity. As expected, Lin-Ub was decreased in *Hoip^A-KO^* WAT as compared to their littermate controls regardless of the diet. Moreover, *Hoip^A-KO^* WAT displayed decreased perilipin levels suggesting impaired lipid storage (Fig. 1A). Whereas *Hoip^A-KO^* mice gained normal weight under CD, they failed to gain weight under HFD, reaching a plateau at 8 weeks of diet (Fig. 1B). All mice displayed comparable food intake and caloric expenditure (Fig. 1C and fig. S1C).

During HFD feeding, metabolism acutely shifts from circadian (alternating carbohydrate and fat usage) to a predominance of fat utilization (*43*). This switch requires higher oxygen consumption for β-oxidation but has a rather constant CO_2_ production, which translates into a lower respiratory exchange ratio (RER) (*43*, *44*). HFD-fed *Hoip^A-KO^* mice showed similar RER as compared to littermate controls (Fig. 1D and fig. S1C), indicating a normal metabolization of both fatty acids and carbohydrates during light and dark circles. Thus, altered energy expenditure cannot explain the difference in body weight gain between HFD groups. In contrast, *Hoip^A-KO^* CD-fed mice showed a decrease in RER during light cycles (Fig. 1D and fig. S1C), suggesting that during reduced-food intake periods, *Hoip^A-KO^* mice preferably use fatty acids as fuel.

Despite less weight gain, *Hoip^A-KO^* mice displayed elevated normal-fed and fasting glycemia under both CD- and HFD-fed conditions (Fig. 1, E and F). Moreover, *Hoip^A-KO^* mice presented increased blood levels of insulin and HbA1c, a surrogate marker for diabetes onset, upon HFD (Fig. 1, G and H). We further studied glucose tolerance at two different time points of HFD feeding (8 and 16 weeks). *Hoip^A-KO^* mice were glucose intolerant in an oral glucose tolerance test (oGTT) at 16, but not at 8, weeks of HFD consumption (Fig. 1I and fig. S1D). Yet, *Hoip^A-KO^* mice were insulin resistant, as evidenced by the insulin tolerance test (ITT) both at 8 and 16 weeks of diet, suggesting a progressive development of metabolic syndrome upon HFD (Fig. 1J and fig. S1E). Notably, *Hoip^A-KO^* mice under CD were not insulin resistant (fig. S1, F and G). Together, our results show that *Hoip^A-KO^* mice exhibit basal hyperglycemia and develop insulin resistance and glucose intolerance upon HFD.

### Adipocyte-specific HOIP deficiency causes lipodystrophy and impaired adipose tissue expansion under HFD

**Fig. 2.**
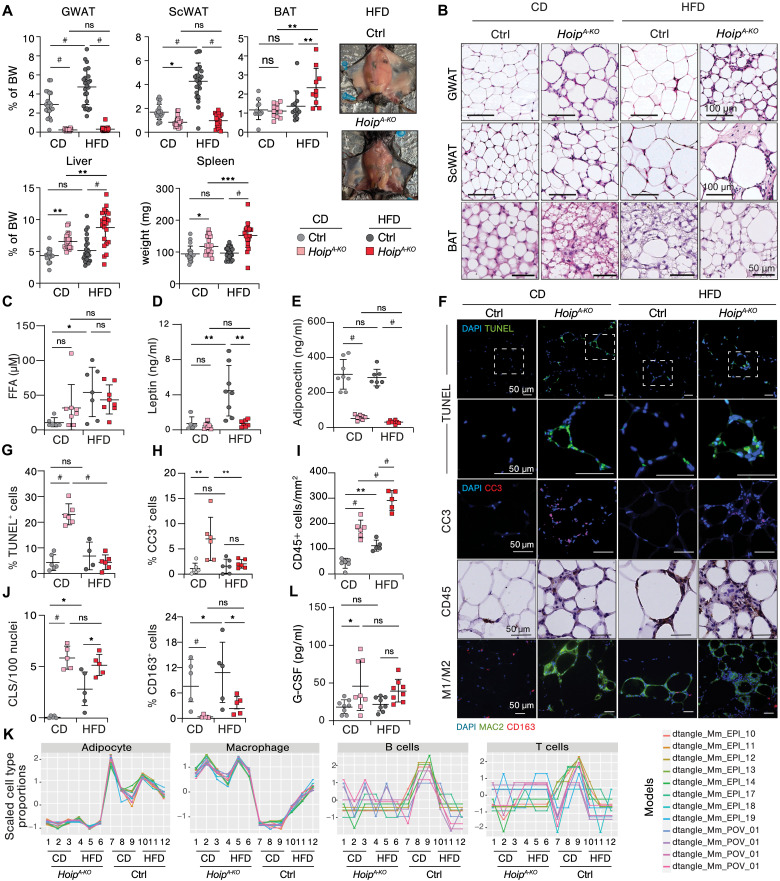
Adipocyte-specific HOIP deficiency causes lipodystrophy and impaired adipose tissue expansion under HFD. (**A**) Tissue weight relativized to total body weight, except spleen (mg) and representative pictures of mice at 16-week diet (*n* = 20 to 24, except BAT: 10 to 13). (**B**) Representative H&E images of GWAT, ScWAT, and BAT at 16-week diet. (**C** to **E**) Serum values at 16-week diet (*n* = 6 to 8) of free fatty acids (FFAs) (C), adiponectin (D), and leptin (E). (**F**) Representative pictures of immunofluorescence and immunohistochemistry of GWAT for the indicated targets at 16-week diet. (**G** to **J**) Quantification of immunostainings in (F) (*n* = 4 to 6): Percentage of dead cells (TUNEL^+^ cells) (G); percentage of cleaved caspase-3 (CC3)–positive cells (H); immune cells (CD45^+^) per mm^2^ of tissue (I); Crown-like-structure (CLS) count (J) and percentage of M2-like macrophages (CD163^+^ cells). (**K**) Deconvolution of bulk RNA-seq analysis of GWAT after 16-week diet (*n* = 2 to 3 pools of three mice) using cell type–specific gene signatures as models for the indicated cell types. (**L**) Granulocyte colony-stimulating factor (G-CSF) serum values at 16-week diet (multiplex). All values represent the means ± SD. Statistics: One-way ANOVA with selected pair comparison, followed by with Bonferroni correction, *P* values GP: #*P* < 0.0001, ***P < 0.0002, **P < 0.0021, and *P < 0.0332; ns, *P* > 0.0333.

To gain a deeper understanding of the metabolic complications in *Hoip^A-KO^* mice, we assessed the different AT compartments histologically. While HFD feeding for 16 weeks increased WAT mass, including subcutaneous (Sc) and gonadal (G) WAT in control animals, this increase was markedly impaired in *Hoip^A-KO^* mice under HFD (Fig. 2A and fig. S2A). In contrast, brown AT (BAT) mass was markedly augmented upon 16 weeks of HFD in *Hoip^A-KO^* mice as compared to littermates (Fig. 2A). Shorter feeding period of 8 weeks also caused a reduction in GWAT and ScWAT but did not affect BAT mass (fig. S2B). Concomitant with reduced WAT mass, *Hoip^A-KO^* mice showed hepatomegaly and splenomegaly upon 8 and 16 weeks of HFD (Fig. 2A and fig. S2, A and B). Notably, WAT mass was also reduced in CD-fed *Hoip^A-KO^* mice, although only GWAT was readily reduced at 8 weeks of HFD feeding. Histological assessment revealed that the WAT presented structural abnormalities, including evidence of immune cell infiltration and extracellular matrix deposition (Fig. 2B). During tissue remodeling in obesity, ScWAT tends to expand via an increase in adipocyte numbers (hyperplasia), while GWAT adipocytes tend to enlarge (hypertrophy) (*45*). Both *Hoip^A-KO^* GWAT and ScWAT displayed highly heterogeneous adipocyte sizes, including hypertrophic adipocytes under CD and failed expandability upon HFD in GWAT (Fig. 2B and fig. S2C). BAT also presented adipocyte hypertrophy, whitening, and inflammation in *Hoip^A-KO^* upon HFD feeding (Fig. 2B). Strikingly, despite a clear lipodystrophy-like phenotype, we did not detect significant dyslipidemia in the serum of *Hoip^A-KO^* mice (Fig. 2C and fig. S2D). Moreover, we observed a down-regulation of lipolysis, adipokines, and adipogenesis-related genes by RNA sequencing (RNA-seq) analysis of GWAT of *Hoip^A-KO^* mice (fig. S2E), suggesting GWAT dysfunction. In normal conditions, the secretion of the adipokines, leptin, and adiponectin by adipocytes is inversely correlated, being increased and reduced, respectively, in response to obesity (*46*). In line with reduced weight gain, mutant mice presented a significant impairment in leptin production in HFD conditions (Fig. 2D). Moreover, we observed a profound and diet-independent suppression of adiponectin secretion (Fig. 2E). This shows that loss of fat mass is accompanied by loss of endocrine function in *Hoip^A-KO^* mice. Collectively, we provide evidence that loss of HOIP leads to spontaneous lipodystrophy with impaired AT expansion and severe perturbation of AT endocrine function under diet-induced obesity.

### Adipocyte death and inflammation spike in adipose tissue with HOIP deficiency

Next, we studied how HOIP deficiency affected cell death responses in GWAT upon HFD. Cell death, as detected by TUNEL (terminal deoxynucleotidyl transferase–mediated deoxyuridine triphosphate nick end labeling) and cleaved caspase-3 stainings, was exacerbated in *Hoip^A-KO^* GWAT under both diets (Fig. 2F). Yet, only under CD this increase was significant (Fig. 2, G and H, and fig. S3A). The lack of quantifiable difference in TUNEL or cleaved caspase-3 stainings between HFD-fed control and *Hoip^A-KO^* mice in GWAT is likely due to the complete disruption of cellular nuclei [e.g., 4′,6-diamidino-2-phenylindole (DAPI) negative] despite clear positive staining and/or dilution of remaining positive cells due to high cellularity in *Hoip^A-KO^* mice. The HOIP depletion in adipocytes induced significant, CD45-positive, immune cell infiltration, predominantly F4/80-positive macrophages, in both CD and HFD conditions (Fig. 2, F and I, and fig.S3B). This infiltration together with significant loss of nuclear integrity makes quantification of standard cell death markers particularly challenging at 16 weeks of HFD. Another proxy of dead adipocytes is the presence of crown-like structures (CLSs). CLSs are composed of M1-like macrophages and coincides with a reduction in resident M2-like macrophages (*47*, *48*). GWAT of *Hoip^A-KO^* mice exhibited a marked increase in CLS formation compared to control mice (Fig. 2, F and J). Conversely, the population of CD163-positive cells (M2-like macrophages) was significantly reduced irrespective of dietary conditions (Fig. 2, F and J).

Last, cell-type deconvolution analysis from bulk RNA-seq of GWAT derived from the different groups revealed a specific depletion of adipocytes across both diets, suggesting that these are the primary cells undergoing cell death (Fig. 2K and fig. S3B). Consistent with our immunohistological analysis, macrophages were the most abundant immune cell infiltrate in *Hoip^A-KO^* mice. The infiltration of other immune cells did not seem to be greatly affected (Fig. 2K and fig. S3C). Macrophage infiltration appeared to be pro-inflammatory as we detected an up-regulation of pro-inflammatory genes in GWAT RNA-seq analysis (fig. S3D). This prompted us to explore how HOIP deficiency affected NF-κB/MAPK activation in GWAT. Western blot analysis revealed increased phosphorylation of NF-kappa-B inhibitor alpha (IκBα) along with degradation of IkBα in *Hoip^A-KO^* GWAT, which was further exacerbated by HFD in both control and HOIP-deficient mice (fig. S3E). Gene set enrichment analysis (GSEA) also revealed that both NF-κB and MAPK pathways were up-regulated upon deficiency of HOIP in adipocytes (fig. S3F). The surge of proinflammatory macrophages, and decay of adipocytes, leads us to speculate that NF-κB/MAPK activation may come from macrophages rather than from adipocytes themselves. Unexpectedly, despite the notable local inflammatory response, only a slight induction of inflammatory cytokines in the serum of *Hoip^A-KO^* mice was observed (fig. S3G), and from the group of tested cytokines, only granulocyte colony-stimulating factor (G-CSF) (Fig. 2L) was significantly elevated upon loss of HOIP under CD. Therefore, our data indicate that loss of HOIP unleashes cell death and exacerbates local inflammation in GWAT without causing systemic inflammation.

### MASLD is exacerbated by HOIP deficiency in adipocytes

**Fig. 3.**
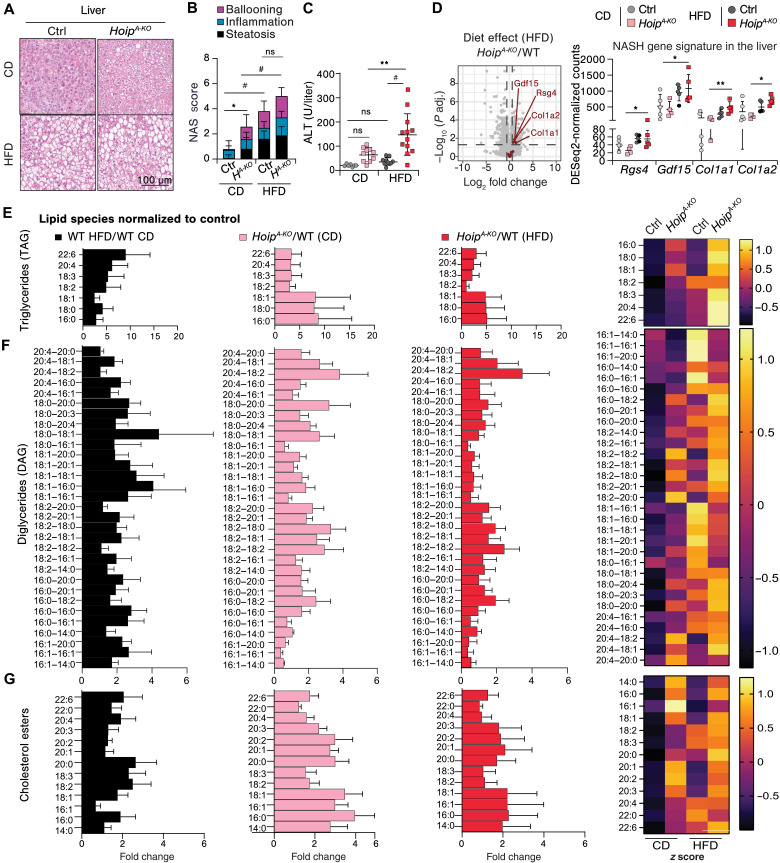
MASLD is exacerbated by HOIP deficiency in adipocytes. Liver evaluation of male mice after 16-week diet: (**A**) Representative H&E images, (**B**) steatosis score (*n* = 14 to 17) of mice evaluated in (A). (**C**) Serum levels of ALT (*n* = 6 to 12). (**D**) Left: Volcano plot of liver RNA-seq data comparing selected NASH signature genes between *Hoip^A-KO^* and control mice on a HFD. The *x* axis represents log_2_ fold change (*Hoip^A-KO^*/WT), and the *y* axis shows −log_10_ (adjusted *P* value) (*P*adj). Red points indicate NASH signature genes significantly up-regulated in *Hoip^A-KO^* mice. Right: DESeq2-normalized counts for the four highlighted NASH-associated genes. (**E** to **G**) Liver lipidomic (*n* = 5) showing triglycerides grouped by first fatty acid (E), diglycerides (F), and cholesterols (G). Trees are expressed as relative to the corresponding control and heat maps correspond to *z* scores of the mean of total lipid species: black: WT HFD/WT CD; pink: CD *Hoip^A-KO^*/WT; red: HFD *Hoip^A-KO^*/WT. Statistics: [(B) and (C)] One-way ANOVA with selected pair comparison, followed by with Bonferroni correction, *P* values GP: #*P* < 0.0001, ***P < 0.0002, **P < 0.0021, and *P < 0.0332; ns, *P* > 0.0333. (D) Log_2_ fold changes and *P* values were obtained using a Wald test with Benjamin-Hochberg correction for multiple testing. *P* values: **P < 0.01 and *P < 0.05.

We next investigated whether loss of HOIP in adipocytes affected the response of peripheral metabolic organs to HFD. As compared to littermate controls, HFD-fed *Hoip^A-KO^* mice did not present intestinal abnormalities (fig. S4, A and B). Despite the observed splenomegaly, supporting an ongoing inflammatory response, there were no major structural abnormalities in the spleen of *Hoip^A-KO^* mice fed with CD or HFD (fig. S4A). In line with the observed hyperinsulinemia, HFD-fed *Hoip^A-KO^* mice suffered from pancreatic islet hyperplasia with increased area of insulin positivity (fig. S4, C to E). Notably, and consistently with hepatomegaly, *Hoip^A-KO^* mice revealed liver steatosis both under CD and HFD feeding for 16 weeks (Fig. 3, A and B, and fig. S5A). Yet, only in response to HFD, this was translated into liver damage reflected by increased serum levels of alanine aminotransferase (ALT) and mildly elevated lactate dehydrogenase (LDH), albumin, and aspartate aminotransferase (AST) (Fig. 3C and fig. S5B). Moreover, liver bulk RNA-seq analysis showed significantly higher expression of MASLD/nonalcoholic steatohepatitis (NASH) signature genes (*49*) in *Hoip^A-KO^* mice, exclusively when fed with a HFD (Fig. 3D and fig. S5C).

To assess the quantity and quality of lipid deposits in the liver, we performed a lipidomic screen. We observed significantly elevated levels of triacylglycerides [triacylglycerol (TAG)], diacylglycerides [diacylglycerol (DAG)] and cholesterol esters (CEs) under CD in *Hoip^A-KO^* mice (Fig. 3, E to G, and fig. S5D). Notably, *Hoip^A-KO^* livers displayed high levels of saturated and monounsaturated fatty acids (MUFAs) regardless of the diet but accentuated by HFD, whereas in control mice, HFD resulted in deposition of mainly polyunsaturated fatty acids (PUFAs). These data revealed that mice lacking HOIP in adipocytes not only suffer from an overall lipid accumulation in the liver, but display a lipid species signature associated with the onset of MASLD (*50*, *51*). Given this observation, we tested lipotoxicity by looking at cell death signatures in liver RNA-seq data. We observed a clear, and almost exclusive, apoptotic signature in HFD-fed *Hoip^A-KO^* mice but not in CD-fed *Hoip^A-KO^* (fig. S5E). This observation further shows that although *Hoip^A-KO^* mice have increased steatosis in basal conditions, liver damage only occurs upon a HFD challenge. Collectively, we provide evidence that linear ubiquitination in adipocytes is essential to prevent MASLD during obesity.

### HOIP deficiency in adipocytes results in an obesogenic transcriptional signature and predisposes mice to aging-related metabolic syndrome

To explore the nature of the metabolic dysfunction observed in *Hoip^A-KO^* mice, we performed bulk RNA-seq of GWAT derived from *Hoip^A-KO^* mice, or littermate controls, under 16 weeks of CD or HFD, as described above. More than 3000 genes were differentially expressed in GWAT of *Hoip^A-KO^* mice versus littermate controls, regardless of the diet. Gene Ontology (GO) enrichment analysis for biological processes (BPs) of negatively regulated genes in *Hoip^A-KO^* GWAT pointed toward defects in metabolic pathways (fig. S6A). This analysis revealed a decrease in the expression of genes associated with response to insulin, lipid metabolism, thermoregulation, and cellular respiration (fig. S6B). In line with our histoimmunological assessment (Fig. 2), biological processes up-regulated in *Hoip^A-KO^* GWAT were mainly related to inflammation, extracellular matrix remodeling, and cell death with genes associated with cytokine production and immune responses, being markedly up-regulated (fig. S7, A and B). GSEA revealed that apoptosis and pyroptosis were significantly enhanced upon HOIP deficiency and HFD feeding, respectively, whereas the enrichment of necroptosis and ferroptosis was not significant (fig. S7C). We then went more in detail and examined the fold change of specific genes that are directly implicated in cell death execution. Genes involved in pyroptosis were highly up-regulated both by HFD and by HOIP deficiency under CD (fig. S7D). However, pro-apoptotic genes were specifically up-regulated by the deletion of HOIP but not by the diet itself. A similar observation was performed with MLKL (fig. S7D).

**Fig. 4.**
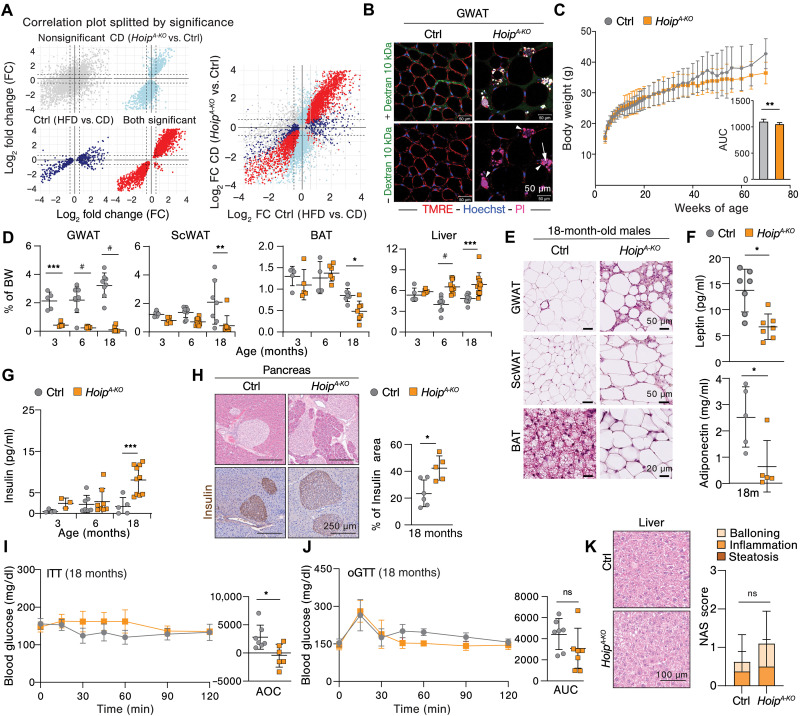
HOIP deficiency in adipocytes predisposes mice to aging-related metabolic syndrome. (**A**) Correlation plots from RNA-seq of mice after 16-week diet showing significant genes for the following comparison are indicated in color: light blue CD (*Hoip^A-KO^* versus WT), blue WT (CD versus HFD), and red both CD (*Hoip^A-KO^* versus WT) and WT (CD versus HFD). (**B**) Representative intravital two-photon microscopy image from live GWAT of 12-week-old male mice following the tail vein injection of fluorescent probes: Red: TMRE (mitochondrial membrane potential), blue: Hoechst (nuclei), pink: PI (cell death), and green: dextran (blood flow). (**C**) Body weight and mean AUC of the resulting curve of mice fed with NCD (*n* = 10 to 12). (**D**) Organ weights relativized to the individual total body weight (*n* = 5 to 12). (**E**) Representative H&E images of GWAT, ScWAT, and BAT of 18-month-old mice. (**F**) Serum levels of leptin and adiponectin of 18-month-old mice (*n* = 5 to 7). (**G**) Serum insulin values during aging (*n* = 4 to 9). (**H**) Representative H&E images and insulin staining of pancreatic islets and quantification of islet area to total tissue of 18-month-old mice (*n* = 5 to 6). (**I**) ITT of 18-month-old matched animals (*n* = 6 to 7). (**J**) oGTT of 18-month-old matched animals (*n* = 6 to 7). (**K**) Representative H&E images of 18-month-old liver and its steatosis score. All values represent the means ± SD. Statistics: (A) Pearson correlation of log_2_ fold changes between comparisons. Genes were grouped by significance, and correlations were calculated within each group (*r* = 0.77 to 0.94). [(C), (F), (H), (I), and (J)]: *t* test (two groups): *P* values: **P < 0.01 and *P < 0.05. [(D, (G), and (K)]: Two-way ANOVA of mutation (grouped by age or score category), with Bonferroni correction, *P* values GP: #*P* < 0.0001, ***P < 0.0002, **P < 0.0021, and *P < 0.0332; ns, *P* > 0.0333.

As HOIP deficiency elicits a spectrum of intrinsic abnormalities in WAT under CD conditions, we speculated that *Hoip^A-KO^* mice exhibit an inherently obesogenic transcriptional profile. To investigate this, we compared the gene signature of the most significantly up- and down-regulated genes in (i) *Hoip^A-KO^* CD versus wild type (WT) CD (light blue) and (ii) WT HFD versus WT CD (dark blue) (Fig. 4A). This comparison revealed a linear correlation (red) between the changes in expression in both groups, suggesting that indeed the absence of HOIP in adipocytes transcriptionally resembles obese, WT, adipocytes (Fig. 4A). Yet, despite the presence of marked hypertrophic adipocytes, aberrant cell death rates, and consequent M1-like CLS (*7*), no signs of metabolic syndrome, such as insulin resistance or MASLD, were observed in young mice in CD conditions (Figs. 1 and 3). These results suggest that HOIP deficiency predisposes to metabolic syndrome in animals under basal conditions, most likely by priming macrophages toward an obesogenic phenotype independently of dietary fat intake. Live imaging by two-photon microscopy revealed spontaneous cell death by propidium iodide (PI) incorporation in hypertrophic adipocytes and concomitant CLS formation in *Hoip^A-KO^* GWAT under normal chow diet (NCD) readily at 3 months of age (Fig. 4B and movies S1 and S2). Macrophages in CLS retain mitochondrial activity [tetramethyl rhodamine, ethyl ester (TMRE) positivity] while also becoming PI positive, which suggests that the engulfment of adipocyte blebs may contribute to the obesogenic gene signature (Fig. 4B and movies S1 and S2).

Based on this, we explored whether HOIP deficiency was sufficient to cause aging-related metabolic syndrome under NCD containing a standard fat content of 9% and studied the physiological consequence of basal adipocyte death through life span. Body weight gain reached a plateau in *Hoip^A-KO^* mice at 40 weeks of age, whereas littermates continued to gain weight over time (Fig. 4C and fig. S8A). Yet, HOIP deficiency in adipocytes did not affect normal glycemia throughout life span until 80 weeks of age (fig. S8B). Aged *Hoip^A-KO^* mice presented a similar phenotype to young mice fed with CD, with decreased GWAT mass starting as early as 3 months of age, followed by loss of ScWAT mass, hepatomegaly, and splenomegaly at 6 months of age (Fig. 4D and fig. S8, C and D). In line with the loss of fat mass, *Hoip^A-KO^* mice displayed WAT hypertropia and whitening of BAT (Fig. 4E), although without dyslipidemia or hyperglycemia at 18 months of age (fig. S8, E and F). The hormonal analysis of these mice further revealed impaired leptin and adiponectin secretion (Fig. 4F) and increased insulin in blood (Fig. 4G). Although old *Hoip^A-KO^* presented pancreatic islet hyperplasia, assessed by hematoxylin and eosin (H&E) and insulin staining (Fig. 4H) and insulin resistance (Fig. 4I), they were not glucose intolerant (Fig. 4J). This, together with the observed normoglycemia and normal Hb1Ac levels, shows that *Hoip^A-KO^* mice are not diabetic but show a prediabetic signature. Moreover, HOIP deficiency in adipocytes did not exacerbate liver steatohepatitis (Fig. 4K and fig. S8, G and H). We next enquired whether other secondary metabolic disorders besides diabetes and MASLD could affect elderly *Hoip^A-KO^* mice, such as cardiovascular defects (*52*) or compromised kidney function (*53*). We could not detect differences in stroke volume, effusion fraction (EF), or end-diastolic volume except for a slight increase in the heart rate in *Hoip^A-KO^* mice (fig. S8I), indicative of preserved cardiac health. Conversely, old *Hoip^A-KO^* mice may be at risk of developing kidney failure, as evidenced by elevated urea in blood (fig. S8J). In summary, these results suggest that HOIP deficiency in adipocytes predisposes mice to develop metabolic complications during aging but is not sufficient to induce diabetes or MASLD unless challenged with HFD.

### HOIP does not regulate adipogenesis in human adipocytes, but it is required for optimal NF-κB activation and protection from cell death upon TNF stimulation

**Fig. 5.**
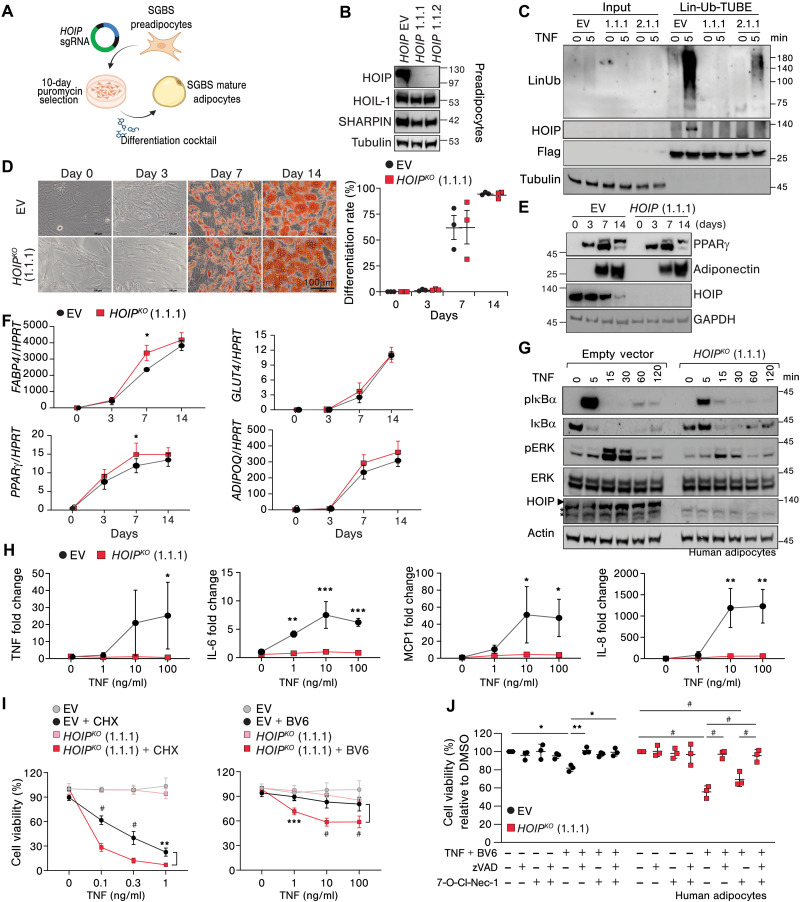
HOIP does not regulate adipogenesis in human adipocytes, but it is required for optimal NF-κB activation and protection from cell death upon TNF stimulation. (**A**) Schematic workflow of human preadipocyte (PA) gene editing and differentiation. (**B**) Western blot of indicated proteins in CRISPR HOIP-KO PA clones; EV, empty vector. (**C**) Lin-Ub TUBE pulldown of mature adipocytes (AP) after TNF (200 ng/ml). (**D**) ORO staining and quantification during differentiation (*n* = 3). (**E**) Western blot of indicated proteins at differentiation stages (*n* = 3). (**F**) qPCR of adipogenesis markers at differentiation stages, normalized to HPRT (*n* = 3). (**G**) AP lysate Western blot of the indicated proteins after TNF (100 ng/ml; *n* = 3). (**H**) qPCR of NF-κB target genes in AP with increasing TNF, normalized to HPRT and relativized to EV (*n* = 3). (**I** to **J**) Percentage of viability after increasing TNF with CHX (50 μg/ml), BV6 (500 nM), or DMSO for 24 hours (*n* = 3) (I) or after 24 hours of TNF (0.1 ng/ml) with inhibitors (*n* = 5) (J). Values presented as means ± SD. Statistics: [(D) to (I)]: Two-way ANOVA grouped by concentration or time including Bonferroni correction. (J) One-way ANOVA of selected treatments with Bonferroni correction. *P* values: #*P* < 0.0001, ***P < 0.0002, **P < 0.0021, and *P < 0.0332. ERK, extracellular signal–regulated kinase. pERK, phosphorylated extracellular signal–regulated kinase.

Next, we aimed to unravel the underlying mechanism by which loss of HOIP in adipocytes causes metabolic dysfunction. We used preadipocyte-human Simpson-Golabi-Behmel syndrome (SGBS) cells as a model system (*54*). We noted that HOIP expression was up-regulated in the initial phase of differentiation toward adipocytes but decreased to baseline levels once adipocytes reached full differentiation (fig. S9A). Given that LUBAC promotes optimal NF-κB activation and that numerous studies highlight the critical role of NF-κB activation in adipogenesis (*55*, *56*), we investigated whether HOIP and Lin-Ub play a role in adipogenesis. To this end, we generated HOIP knockout (KO) preadipocytes by CRISPR-Cas9 (Fig. 5, A and B, and fig. S9B). As expected, HOIP-KO preadipocytes could not generate Lin-Ub chains upon TNF stimulation (Fig. 5C). After 7 days of adipogenic differentiation, both HOIP-KO and WT preadipocytes showed lipid droplet formation at a comparable rate as shown by Oil Red O (ORO) staining (Fig. 5D). The protein expression of peroxisome proliferator–activated receptor γ (PPARγ), a crucial regulator of adipogenesis (*57*), and adiponectin was induced in mature adipocytes regardless of the presence of HOIP (Fig. 5E). In addition, the expression of adipocyte differentiation markers *GLUT4* and *ADIPOQ* was comparably up-regulated in KO and WT cells during the differentiation process, with a slight increase in the adipogenic genes *PPAR*γ and *FABP4* at 7 days, which was normalized at 14 days (Fig. 5F).

Because of the established role of HOIP in regulating the balance between NF-κB activation and cell death upon TNF stimulation, we next evaluated whether these processes were affected in adipocytes by the absence of HOIP. Contrary to what we observed in whole GWAT extracts (fig. S3, D and E), we found that HOIP deficiency led to a markedly reduced NF-κB and MAPK activation upon TNF stimulation in both mature and preadipocytes (Fig. 5G and fig. S9C). In line, the expression of NF-κB target genes such as IL-6, Monocyte Chemotactic Protein-1 (MCP1), also known as Chemokine (C-C motif) ligand 2 (CCL2), IL-8, and TNF itself was blunted in cells lacking HOIP and treated with TNF (Fig. 5H and fig. S9D). TNF has been described to promote expression of adiponectin and leptin and to be important for adipogenesis (*9*, *58*). Yet, no effect was observed on the expression of adipogenic markers in mature adipocytes upon TNF stimulation or HOIP loss (fig. S8E). These data show that HOIP loss specifically impairs NF-κB/MAPK activation in adipocytes upon TNF stimulation and implies that indeed increased NF-κB/MAPK signature in whole tissue lysates probably originates from infiltrating macrophages.

HOIP deficiency did not alter sensitivity to TNF-induced cell death alone in adipocytes, consistent with previous observations in primary human fibroblasts (*38*). Yet, it significantly sensitized both mature and preadipocytes to death upon stimulation with TNF and cycloheximide (CHX) or the cIAP1/2 inhibitor, SMAC mimetic (BV6) (see fig. S9F) in a dose-dependent manner (Fig. 5I and fig. S9G). Intriguingly, cell death was prevented by treatment with the pan-caspase inhibitor zVAD-fmk (zVAD) with no additional effect of the RIPK1 kinase inhibitor (Nec-1) in HOIP-deficient adipocytes (Fig. 5J and fig. S9, H and I). This suggests that human adipocytes lacking HOIP mainly die by RIPK1-independent apoptosis. In summary, our results indicate the lack of Lin-Ub does not interfere with adipogenesis but rather dampens NF-κB/MAPK activation and sensitizes cells to apoptosis upon TNF stimulation in primary human mature and pre-adipocytes.

### Caspase-8–mediated cell death is responsible for metabolic dysfunction driven by HOIP deficiency

Next, we aimed to address whether increased cell death was responsible for spontaneous lipodystrophy in *Hoip^A-KO^* mice. Despite the finding that human adipocytes mainly undergo caspase-dependent cell death with no clear induction of necroptosis upon caspase inhibition, mouse mature 3T3-L1 adipocytes were able to undergo both apoptosis and necroptosis in vitro under TNF stimulation (fig. S10 A and B). To assess whether caspase-dependent cell death was responsible for spontaneous lipodystrophy in *Hoip^A-KO^* mice, we codeleted caspase-8 in mature adipocytes by crossing *Casp8^A-KO^* with *Hoip^A-KO^* mice (hereafter *Casp8^A-KO^Hoip^A-KO^*), and since deletion of caspase-8 inactivation may unleash necroptosis in mouse cells, we additionally deleted MLKL and evaluated them under NCD.

**Fig. 6.**
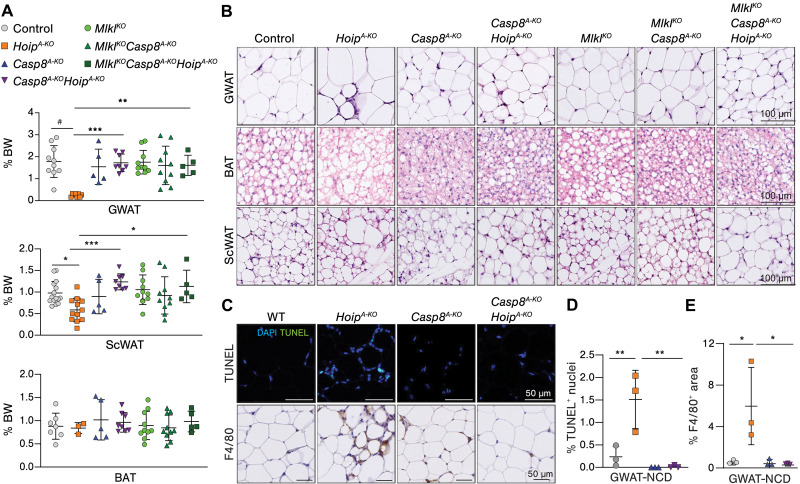
Caspase-8–dependent cell death underlies spontaneous lipodystrophy in adipocyte-specific HOIP-deficient mice. (**A**) Tissue weights relativized to total body weight (*n* = 5 to 12) of 12-week-old male mice fed with NCD. (**B**) Representative H&E pictures of 12-week-old male mice. (**C**) Representative pictures of different stainings of GWAT: TUNEL (dead cells) and F4/80 (macrophages). (**D** and **E**) Quantification of the stainings in (C): Percentage of TUNEL-positive nuclei (*n* = 3) (D) and F4/80-positive area normalized to total area (E). Values presented as means ± SD. Statistics: One-way ANOVA of selected comparison with Bonferroni correction. *P* values: #*P* < 0.0001, ***P < 0.0002, **P < 0.0021, and *P < 0.0332.

No differences in weight gain or normal-fed glycemia (fig. S10C) were found between *Hoip^A-KO^*, *Casp8^A-KO^Hoip^A-KO^*, and *Mlkl^KO^-Casp8^A-KO^Hoip^A-KO^* mice and respective littermate controls (N.B. WT, *Casp8^A-KO^* and *Mlkl^KO^* and *Mlkl^KO^Casp8^A-KO^* mice) at 12 weeks of age. Notably, the deletion of caspase-8 in adipocytes was sufficient to completely restore fat mass in *Hoip^A-KO^* mice (Fig. 6A). Moreover, caspase-8 deletion fully prevented disruption of WAT architecture without clear contribution of MLKL deletion (Fig. 6B). Moreover, BAT whitening was prevented by caspase-8 loss in *Hoip^A-KO^* mice, and both *Casp8^A-KO^* and *Casp8^A-KO^Hoip^A-KO^* BAT showed accumulation of smaller lipid droplets (Fig. 6B). No defects in any other organs analyzed were observed at 12 weeks of age (fig. S10, D and E). Last, cell death and macrophage infiltration observed in *Hoip^A-KO^* mice were fully prevented by loss of caspase-8 (Fig. 6, C to E). Collectively, we show that caspase-8 deficiency in adipocytes prevents cell death caused by loss of HOIP and does not unleash necroptosis in NCD conditions. Although this contradicts our in vitro data using mouse cells, it goes in line with our findings in human adipocytes (Fig. 5) and a recent publication (*11*). In turn, the prevention of caspase-8–dependent cell death prevents lipodystrophy caused by the absence of HOIP.

**Fig. 7.**
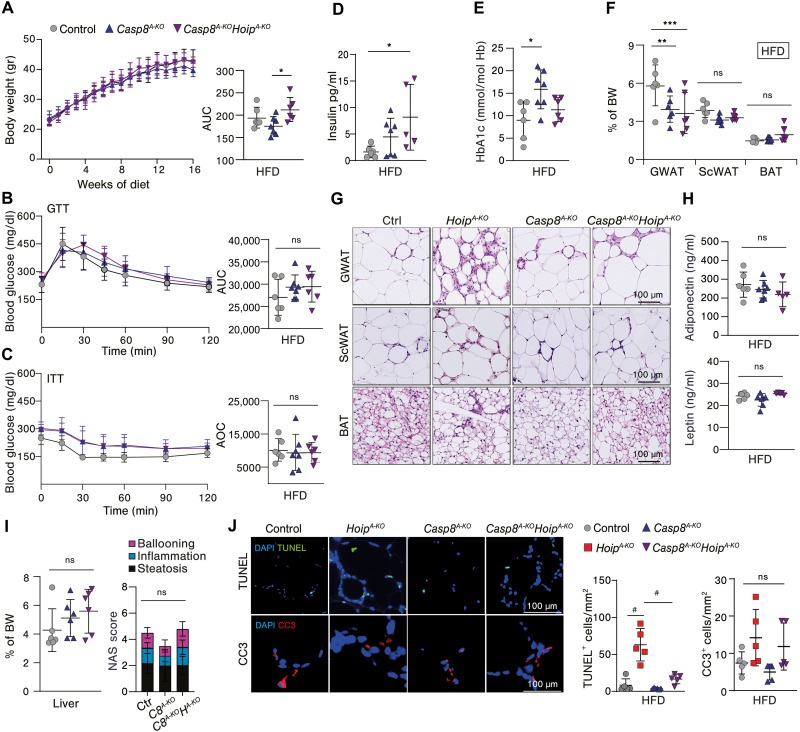
Caspase-8–mediated cell death is responsible for metabolic dysfunction driven by HOIP deficiency in adipose tissue. (**A** to **F**) Evaluation of mice with indicated genotypes after 16-week HFD (*n* = 5 to 7). (A) Weight monitoring curve and individual AUC (B), oGTT and individual AUC (C), and ITT and individual AOC. (D) Insulin serum values after 16-week-HFD of insulin. (E) Blood HbA1c levels. (F) Tissue weights relative to total body weight. (**G**) Representative H&E pictures of WAT and BA. (**H**) Adipokinese serum valus (*n* = 5 to 7). (**I**) Liver steatosis score (*n* = 5 to 7). (**J**) Representative pictures of immunoflourescence staining of GWAT (*n* = 5): TUNEL and cleaved caspase-3 (CC3) and respective quantification (positive cells per area). Values presented as mean ± SD. Statistics: One-way ANOVA of selected genotype comparisons with Bonferroni correction. *P* values: #*P* < 0.0001, ***P < 0.0002, **P < 0.0021, and *P < 0.0332; ns, *P* > 0.0333.

To explore whether *Casp8^A-KO^Hoip^A-KO^* mice develop metabolic syndrome upon obesity, we challenged them with HFD. *Casp8^A-KO^Hoip^A-KO^* mice gain weight similarly to their *Casp8^A-KO^* littermate controls (Fig. 7A). There were no significant differences observed among any of the groups regarding normal-fed or fasting glucose levels (fig. S11, A and B), nor in glucose tolerance (Fig. 7B) or insulin sensitivity (Fig. 7C) after 16 weeks of HFD. Although insulin levels were higher in *Casp8^A-KO^Hoip^A-KO^* mice, glycemia did not change, as compared to HFD-fed WT littermate controls, which goes in line with the fact that the mice do not display gross abnormalities in glucose tolerance (Fig. 7, D and E). Fat mass was comparable between obese *Casp8^A-KO^* and *Casp8^A-KO^Hoip^A-KO^* mice, and in both cases, GWAT mass was decreased as compared to control WT mice (Fig. 7F), as previously reported for *Casp8^A-KO^* mice (*11*). Curiously, although fat mass was normal, GWAT hypertrophy, with immune cell infiltration, was only partially prevented in obese *Casp8^A-KO^-Hoip^A-KO^* mice (Fig. 7G). Yet, AT endocrine function in *Casp8^A-KO^Hoip^A-KO^* was not affected as monitored by serum levels of adiponectin and leptin (Fig. 7H). Further analysis on other symptoms of metabolic dysfunction showed no differences in islet size, hepatomegaly, and splenomegaly in *Casp8^A-KO^Hoip^A-KO^* mice and no increased liver damage as compared to controls (Fig. 7I and fig. S11 C to E). Both *Casp8^A-KO^* and *Casp8^A-KO^Hoip^A-KO^* mice showed increased cholesterol in the blood, suggesting slight dyslipidemia likely caused by caspase-8 loss (fig. S11F). The observation that *Casp8^A-KO^Hoip^A-KO^* mice were not fully protected from GWAT hypertrophy upon HFD prompted us to evaluate whether caspase-8–independent cell death was present in these mice. TUNEL-positive staining was fully blocked in HFD-fed *Casp8^A-KO^Hoip^A-KO^* mice, indicating that loss of caspase-8 prevents cell death caused by HOIP deficiency (Fig. 7J and fig. S11G). The differences in the levels of caspase-3 activation between the groups were overall not significantly different (Fig. 7J and fig. S11G). Yet, it appears that loss of caspase-8 did not fully prevent the elevated levels of caspase-3 activation in HFD-fed *Hoip^A-KO^* mice, akin to the residual hypertrophy in these mice (Fig. 7J and fig. S11G). These results highlight the importance of Lin-Ub in protecting against obesity-induced metabolic syndrome primarily through the suppression of caspase-8–dependent cell death.

### HOIP expression correlates with metabolic health likely by modulating cell death responses in patients with obesity

**Fig. 8.**
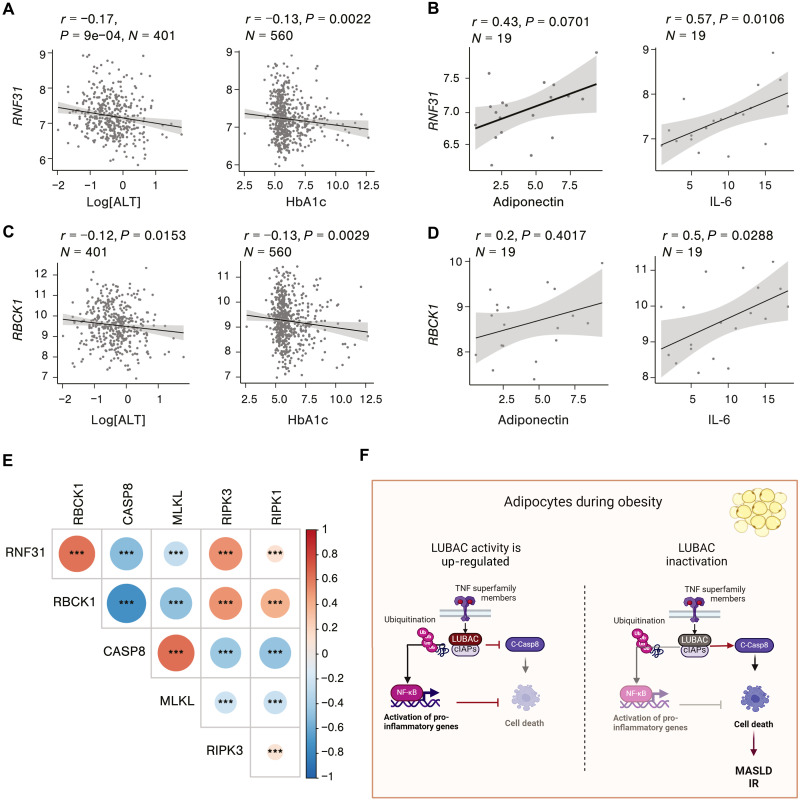
HOIP expression correlates with metabolic health likely by modulating cell death responses in patients with obesity. (**A**) Correlation between RNF31 (HOIP) expression in human visceral adipose tissue (VAT) and serum values of ALT, HbA1c, and sequencing datasets of a cross-sectional cohort. (**B**) Correlation between RNF31 (HOIP) expression in human VAT and serum values of adiponectin and IL-6 from RNA-seq datasets of obese individuals’ cohort. (**C**) Correlation between RBCK1 (HOIL-1) expression in human VAT and serum values of ALT (males), HbA1c (females), and sequencing datasets of a cross-sectional cohort. (**D**) Correlation between RNF31 RBCK1 (HOIL-1) expression in human VAT and serum values of adiponectin (males) and IL-6 (males) from RNA-seq datasets of obese individuals’ cohort. (**E**) Correlation matrix of expression of LUBAC components, RNF31 (HOIP) and RBCK1 (HOIL-1), and cell death effectors analyzed from RNA-seq datasets of VAT cross-sectional cohorts of obese individuals. (**F**) Schematic model of the role of LUBAC in adipocytes to prevent metabolic syndrome during obesity challenge. Statistics: Gender-adjusted analysis was performed, and unless male and female samples were analyzed separately. Median transcript integrity number (TIN) of the data was adjusted, and Spearman coefficient test was performed without multiple testing correction. *P* values: ***P < 0.001.

Next, we investigated the correlation between the expression of RNF31 (HOIP) in visceral (V) adipose tissue from RNA-seq data of the Leipzig Obesity BioBank (LOBB) and various markers of the metabolic syndrome in serum of lean and mainly obese patients collected cross-sectionally. The HOIP expression was negatively correlated with ALT and the diabetic marker HbA1c (Fig. 8A) (*59*). Moreover, in patients suffering from morbid obesity, HOIP is positively correlated with the levels of the anti-inflammatory adipokine, adiponectin, which is associated with metabolic fitness (*60*), and with IL-6 (fig. 87B). The latter was recently found to prevent metabolic syndrome caused by obesity when released by macrophages (*61*). The expression of RBCK1 (HOIL-1) followed a similar pattern (Fig. 8, C and D). We then explored the correlation between LUBAC components, HOIP and HOIL-1, and cell death in the human visceral adipose tissue (VAT) expression analysis. This revealed a notable negative correlation between the expression of LUBAC components and caspase-8 (Fig. 8E), in line with our findings in mouse models in which HOIP prevents caspase-8–driven apoptosis. In addition, MLKL and LUBAC expression were negatively correlated to each other, and although the role of MLKL in AT remains debated, we did not observe signs of necroptosis in our experimental models. The LUBAC expression significantly correlated with RIPK1 and RIPK3 expression. This aligns with the previous report showing that RIPK3 acts as an inhibitor of apoptosis in adipocytes during obesity (*13*). Together, these data suggest that LUBAC expression is positively associated with improved metabolic health in obese individuals most likely by inhibiting caspase-8–mediated cell death.

## DISCUSSION

The results of this study provide critical insights into the inflammatory role of adipocytes in metabolic syndrome by identifying Lin-Ub, as an essential regulator of adipocyte cell death. The genetic deletion of HOIP in adipocytes causes the exacerbation of a range of metabolic disturbances, particularly insulin resistance (IR) and MASLD, under obesity. HOIP deficiency led to hypertrophy and increased cell death in GWAT, accompanied by a marked increase in immune cell infiltration, particularly M1-like macrophages, which are known to exacerbate metabolic dysfunction (*47*, *48*). Despite these significant local effects, systemic inflammation was only mildly elevated. The lack of systemic inflammation raises intriguing questions. It suggests that local AT health, particularly in GWAT, is sufficient to cause IR or MASLD in obesity. This may occur not only due to impaired secretion of adipokines but also caused by a yet undefined cross-talk between AT and other metabolic organs, particularly the liver, that may depend on damage signals secreted by dead adipocytes. The identification of these factors in the future will be of great importance for the design of therapeutic targets against metabolic syndrome. Still, we cannot discard the possibility that the liver may adapt to HOIP loss from birth, potentially compensating by storing lipids. Subsequently, upon HFD, excess dietary lipid uptake saturates hepatocytes and causes MASLD. In this context, our findings would be of particular interest to patients with inherited mutations in genes that affect Lin-Ub or cell death responses (*39*, *62*) or inherited lipodystrophies.

We found that HOIP deficiency predisposes mice to metabolic syndrome, manifesting as a lipodystrophy-like syndrome from a young age. This underscores the critical role of Lin-Ub in metabolic health as a fundamental developmental factor in adipocytes, extending beyond obesity to encompass other conditions such as lipodystrophies. These disorders share clinical features with obesity, including dyslipidemia, IR, and liver steatosis. In addition, they have far-reaching effects on overall health, contributing to premature aging (*63*). The RNA-seq of HOIP-deficient mice showed an obesogenic transcriptional profile, including impaired adipokine expression that suggests loss of adipose tissue endocrine function. We stipulate that the latter is likely a consequence of adipocyte death and loss of fat mass because the expression of adipokines was neither affected by HOIP expression nor by TNF exposure in mature adipocytes ex vivo. However, we cannot exclude that other factors in the context of obesity-induced chronic inflammation may also play a role in adipokine signaling in the absence of HOIP. In summary, LUBAC safeguards adipose tissue health and prevents metabolic disorders. This makes *Hoip^A-KO^* mice a suitable model to study nonautoimmune lipodystrophy, mimicking chronic injury-related wasting.

TNF- or TNFR1-deficient mice are protected from HFD-induced obesity (*64*, *65*). This was mainly attributed to decreased inflammation caused by reduced NF-κB activation upon obesity in the absence of TNF. Yet, because NF-κB–mediated inflammatory gene activation and cell death regulation are highly interlinked processes, their respective contributions to inflammation during metabolic dysfunction remain unknown. This knowledge gap together with the suggested tissue-specific functions of NF-κB during metabolic syndrome (*10*) led to controversies on the reported importance of NF-κB signaling in metabolic homeostasis (*9*). Furthermore, to date, the molecular drivers of death receptor–induced cell death in adipocytes are unknown. For example, adipocytes lacking IKKβ suffer from a similar lipodystrophy-like phenotype and IR upon HFD as *Hoip^A-KO^* mice (*66*, *67*), which is likely caused by exacerbated cell death. Because IKKβ regulates cell death independently of NF-κB activation (*68*), this mutation does not provide clarity on the role of NF-κB–mediated inflammatory gene activation in adipocytes. In another example, dimerization-induced caspase-8 activation in adipocytes promotes metabolic syndrome during diet-induced and genetic obesity (*19*), and although this proves that acute cell death causes lipodystrophy, it does not shed light on the molecular events that control cell death in adipocytes. Here, we show that caspase-8 deletion is sufficient to prevent metabolic dysfunction caused by HOIP deficiency, positioning LUBAC as a key regulator of cell death in adipocytes and in the consequent onset of lipodystrophy and obesity-related metabolic dysfunction (Fig. 8F). Mice lacking adipocyte caspase-8 were reported to show improved thermogenesis and metabolism on HFD due to reduced NF-κB activation, suggesting that NF-κB inhibition may improve metabolic health (*11*). However, we could not observe an improved response to HFD in *Casp8^A-KO^* or in *Casp8^A-KO^Hoip^A-KO^* mice as compared to WT mice in our study. Therefore, it is likely that cell death responses play a major role in triggering inflammation than NF-kB affecting on metabolic processes when LUBAC is disrupted.

Both apoptosis and pyroptosis appeared to be highly enriched in GSEA data of *Hoip^A-KO^* mice, yet caspase-8 deletion was sufficient to prevent lipodystrophy and obesity-associated metabolic syndromes such as IR and MASLD. It is possible to speculate that caspase-8 may be driving both apoptosis and pyroptosis by cleavage of Gasdermin D as previously reported (*69*, *70*). However, the pyroptotic gene signature was enriched in WT mice under HFD conditions as much as in *Hoip^A-KO^* under CD conditions. This suggests that pyroptosis may not be directly regulated by LUBAC expression but rather as a result of primed inflammatory macrophages (*71*). Hence, we conclude that caspase-8 is essentially driving apoptosis in adipocytes.

Noteworthy, although caspase-8 deficiency is sufficient to prevent lipodystrophy and metabolic syndrome in response to HFD, it does not fully prevent HFD-induced GWAT hypertrophy in *Hoip^A-KO^* mice, possibly because of persistent caspase-3 activation, as noted by immunostaining. This result suggests that HOIP regulates intrinsic apoptosis which aligns with our differential expression analysis based on the negative (DEseq) data showing elevated expression levels of Bax and Bak in HOIP-adipocyte deficient mice. However, although this level of caspase-3 activation may drive local inflammation, it does not seem to further contribute to metabolic complications associated with HFD feeding (as compared to littermate controls). Further investigation is necessary to understand the consequences of possible sublethal levels of caspase-3 activation in GWAT.

Although the contribution of necroptosis in AT has never been formally proven, there are several reports suggesting that necroptosis inhibition improves the pathological outcome of obesity. For instance, *RIPK1* gene variants were associated with diabetes in humans and its down-regulation ameliorated obesity-driven metabolic syndrome (*12*). Yet, the contradictory reports on the role of MLKL during obesity do not facilitate our understanding of this cell death modality in the modulation of metabolic disorders (*72*–*76*). Although we show that mouse adipocytes can execute necroptosis in vitro, the deletion of caspase-8 did not unleash aberrant necroptosis in vivo despite the loss of HOIP-sensitizing adipocytes to cell death. This could be due to the fact that 3T3-L1 adipocytes are not primary cells but also because we might be using stimuli that do not necessarily recapitulate the complexity of an obese environment in AT. Nevertheless, our results recapitulate the molecular observations in primary human adipocytes because caspase inhibition was indeed sufficient to prevent TNF-induced cell death in the absence of HOIP. Moreover, MLKL deletion had no beneficial impact on the fat mass of *Casp8^A-KO^Hoip^A-KO^* mice. Coupled with the observation that caspase-8 loss prevents cell death, as evidenced by TUNEL staining, in the absence of HOIP under HFD conditions, we conclude that there is either a distinct regulation of the necroptosis machinery in adipose tissue independently of caspase-8 or that adipocytes do not undergo lethal necroptosis at all in the absence of caspase-8. At this stage, we cannot formally discard that necroptosis, perhaps in a sublethal form (*77*), may drive GWAT hypertrophy in *Casp8^A-KO^Hoip^A-KO^* mice.

Severe autoinflammation and recurrent infections have been broadly reported in LUBAC-deficient patients, and several therapies, including TNF inhibition, partially ameliorate some of these symptoms. Yet, how to tackle the metabolic complications of these patients have never been explored. Here, we find an abundance of MUFAs in the liver of *Hoip^A-KO^* mice, regardless of dietary intake, which are usually associated with MASLD. PUFA intake has been linked to the prevention of liver fat accumulation (*78*). Hence, specific dietary conditions such as PUFA-enriched diets may offer benefits against liver steatosis to patients bearing perturbations in Lin-Ub. Further investigation in this direction is not only warranted but imperative for the care of these patients. In a wider population, we show that the expression of LUBAC components in AT is significantly associated with improved metabolic fitness in patients with obesity potentially by preventing caspase-8–dependent cell death. Thus, LUBAC activity, by promoting a healthy AT expansion during obesity, prevents ectopic lipid deposition and, consequently, may serve as a protective mechanism against obesity-associated disorders in humans.

We propose a model in which cytokines, such as TNF, trigger the activation of LUBAC to protect stressed adipocytes from cell death while maintaining sufficient levels of NF-κB activation (Fig. 8F). Under obesity, adipocyte death activates inflammatory macrophage infiltration amplifying the inflammatory response and leading to metabolic syndrome. This cell death is exacerbated when Lin-Ub is low or absent, further boosting inflammation and metabolic dysfunction. Notably, LUBAC may also be important for the homeostatic response to other stress responses in AT during obesity, such as hypoxia. LUBAC has been reported to be crucial in protecting cells against hypoxia-mediated cell death (*79*–*81*).

Collectively, this study demonstrates that LUBAC functions as a protective shield against cell death in adipocytes, thereby defending against metabolic syndrome, particularly MASLD during obesity and potentially, other conditions such as lipodystrophy and aging, placing Lin-Ub as a key metabolic safeguard mechanism.

## MATERIALS AND METHODS

### Animals

*Hoip^A-KO^* and *Casp8^A-KO^* mice were generated by crossing *Hoip*^*fl/f*l^ (*33*) and *Casp^fl/fl^* (*82*), donated by M. Pasparakis, with Adipoq-Cre Strain 010803 [full name: B6;FVB-Tg(Adipoq-cre)1Evdr/J, RRID MSR_JAX:010803, the Jackson Laboratory]. Mice were kept at 22° to 23°C 45% humidity with a light cycle of 12 hours, except for the matched age 72-week-old cohort of *Hoip^A-KO^* kept in a different mouse facility at 25° to 27°C 45% humidity*. Hoip^A-KO^Casp8^A-KO^Mlkl^KO^* mice crossing with the *Hoip^A-KO^Casp8^A-KO^* with *Mlkl^KO^* generated by H.W. (*32*). For all the experiments Cre negative littermates were used as control. Mice in experimental HFD cohorts were fed ad libitum with CD DIO LS–13% fat and 11% sucrose (Ssniff, E15748-04) and HFD DIO–60% (Lard) (Ssniff, E15742-347). Mice under aging cohorts and all mice under maintenance were fed with NCD (9% fat) (Ssniff, V1534). Breeders are fed with breeding chow (15% fat; Sniff, V1154). Cohorts of *Hoip^A-KO^* experiments were bred and performed simultaneously using the same parental line. The designation to diet group was randomized. Five different cohorts were fed to generate the samples used in this study. In all experiments, 6- to 7-week-old male mice were fed with a CD or HFD as indicated for 8 or 16 weeks. Three to five mice were placed per cage, and weight body gain was measured weekly. Normal-fed blood glucose was taken every 3 weeks with an Aida Glucometer and strips (Aida, 781783). At the end of the feeding protocol, mice were euthanized using ketamine/xylazine cocktail [ketamine (100 mg/kg) and xylazine (20 mg/kg)] with a dose of 5 μl/g, this was followed by heart puncture, cervical dislocation, and tissue harvesting. All animal experiments were approved by the Federal Ministry for Nature, Environment and Consumers’ Protection of the state of North Rhine-Westphalia (LANUV/LAVE) and were performed following the respective national, federal, and institutional regulations and following the Replacement, Reduction, and Refinement (3Rs) guidelines under the license numbers: Az: 81-02.04.2020.A349 and Az: 81-02.04.2020.A022.

### In vivo metabolic studies

Glucose tolerance tests were performed on 6-hour fasted animals using glucose (2 g/kg body weight) supplied by oral gavage. ITTs were performed on animals fasted for 6 hours using insulin Lispro (Lilly) injected intraperitoneally. The dose was 0.75 U/kg body weight for HFD-fed mice and 0.5 U/k for mice fed with the CD. Measurements of blood glucose were taken at 0, 15,30, 45, 60, and 120 min after the injection and measured with an Aida Glucometer and strips (Aida, 781783). Mice from three independent experiments were pooled to generate the statistics, except for 8 weeks (only one cohort). For energy expenditure, food, water, and oxygen consumption measurements, mice were individually housed in metabolic cages (TSE PhenoMaster V7.7.3 2021-4898) with free access to food and water after 7 days of training at 22°C and 50% humidity. Data were collected during 72 hours, and the average was expressed by consumption per day and relativized to initial body weight as indicated. Metabolic rate (kcal/day) was calculated using the formula = 1.44 × (3.94 × VO_2_ + 1.11 × VCO_2_) the average of the 3 days was taken as an individual value per mouse. RERs were calculated as VCO_2_/VO_2_ at every single point (every 20 min) during 72 hours. The average in the different phases (total, light, and dark) of each mouse was expressed as a single RER value. Insulin levels were measured by a mouse insulin ELISA kit 90080, CrystalChem Inc.). Serum adipokines were measured by Luminex technology in a custom-made multiplex plate and Adiponectin Luminex Discovery Assay (R&D Systems, LXSASM-01). Blood values Hb1ac, ALT, AST, LDH, urea, and albumin were determined by the UniKlinik chemical laboratory service at the University Hospital. Serum lipid values were determined following the manufacturer’s instructions with the following kits: free fatty acids (Abnova, KA1667), cholesterol (Promega, J3190), and triglycerides (Promega, J3160).

### Cell culture

#### Mouse adipocytes and preadipocytes cell culture

The fibroblast preadipocyte line 3T3-L1-CL-173 (American Type Culture Collection) was kept at 37°C in 5% CO_2_ condition (*83*) on Dulbecco’s modified Eagle’s medium (DMEM) Hi-Glucose (4.5 mg/liter) (Thermo Fisher Scientific, 61965059), 10% calf serum (500 ml; Sigma-Aldrich, 12133C), and 1% penicillin-streptomycin (Pen-Strep) (PAN Biotech, P06-07100). Adipogenic differentiation was induced when cells reached 100% confluency. On day 0, the culture medium was replaced by adipogenic medium I containing DMEM high-glucose 10% fetal bovine serum, 1% Pen-Strep, insulin (1 μg/ml) (10 mg/ml; Sigma-Aldrich, I-9278), dexamethasone (0.25 μM; 100 mg; Sigma-Aldrich, D-2915), 500 μM 3-isobutyl-1-methylxanthin (IBMX; 250 mg; Sigma-Aldrich, I-7018), and 2 μM rosiglitazone (10 mg; Sigma-Aldrich, R2408). On day 3 of adipogenic differentiation, adipogenic medium I was replaced by adipogenic medium II consisting of DMEM high-glucose 10% FBS 1% Pen-Strep and insulin (1 μg/ml), and changed every 2 days, cells were fully differentiated on day 10 of adipogenic differentiation.

#### *SGBS cell culture* (*human cells*)

SGBS cells were derived from the stromal vascular fraction of the subcutaneous adipose tissue from a patient with SGBS (*54*). SGBS cells are maintained in DMEM/F12 containing 33 μM biotin (Sigma-Aldrich), 17 μM pantothenate (Sigma-Aldrich), and 10% fetal calf serum (Gibco, Thermo Fisher Scientific) in a humidified incubator at 37°C with 5% CO_2_. Adipogenic differentiation of SGBS cells was induced when cells reached 90% confluency. On day 0, cells were carefully washed twice with Dulbecco’s phosphate-buffered saline (DPBS) (Gibco). Culture medium was replaced by adipogenic medium I containing DMEM/F12 medium (Gibco) supplemented with 33 μM biotin (SA), 17 μM pantothenate (SA), transferrin (10 μg/ml), 20 nM insulin, 100 nM cortisol, 0.2 nM triiodothyronine (T3), 25 nM dexamethasone, 250 μM IBMX, and 2 μM rosiglitazone. On day 4 of adipogenic differentiation, adipogenic medium I was replaced by adipogenic medium II consisting of DMEM/F12 medium supplemented with 33 μM biotin, 17 μM pantothenate, transferrin (10 μg/ml), 20 nM insulin, 100 nM cortisol, and 0.2 nM T3. SGBS cells are fully differentiated on day 14 of adipogenic differentiation.

### Statistical analysis

Data were analyzed using GraphPad Prism software (version 8.4.1) (San Diego, CA). Area under the curve and area over the curve were calculated as previously described (*84*). Statistical tests were used as described in the figure legends. All data are presented as means ± SD and was analyzed by analysis of variance (ANOVA) with Bonferroni’s post hoc multiple comparison test; in all cases, test were applied to the indicated groups control except for experiments with only two groups in which unpaired individual parametric Student’s *t* test was performed assuming same variation between populations. Correlations were assessed by nonparametric Spearman’s test. Statistical significance was indicated as follows: for *t* test [*P* values: **P < 0.01 and *P < 0.05, not significant (ns)] or one-way or two-way ANOVA with Bonferroni correction for multiple comparisons (*P* values GP: #*P* < 0.0001; ***P < 0.0002; **P < 0.0021; *P < 0.0332; and ns, *P* > 0.0333. In the serum analyses, some results from different longitudinally followed groups of mice were combined. Animals of the same sex and age were used to minimize physiological variability and to reduce SD from the mean. In case of death or sickness, the animal was excluded from the analysis. Tissue samples were excluded in cases of failure in the extraction of RNA or protein of suitable quality and quantity.

### Generation of SGBS empty vector and HOIP KO cells (human preadipocytes)

Knockout SGBS preadipocytes were generated using a CRISPR-Cas9 system. Single guide RNA (sgRNA) duplexes for HOIP (sgRNA1: AACCGCGACATCGAGTCAGT and sgRNA2: GGTGGCACGACGCTTTCTGT) were designed using CRISPick (Broad Institute) and inserted into the pMuLE ENTR U6 stuffer sgRNA scaffold L1-R5 (kindly provided by I. Frew; Addgene #62127; http://n2t.net/addgene:62127; RRID: Addgene_62127) (*85*). Sanger sequencing was used to verify the correct insertion. Using LR Clonase II Plus (Thermo Fisher Scientific), the specific pMuLE ENTR U6-sgRNA and pMuLE ENTR CMV-hCas9 L5-L2 plasmids were recombined with SleepingBeauty transposon plasmid pMuSE eGFP-P2A-PuroR-DEST (*86*). SGBS preadipocytes were cotransfected with the resulting pMuSE U6-sgRNA+CMV-hCas9+RPBSA-eGFP-P2A-PuroR plasmids and the SleepingBeauty-expressing pCMV(CAT)T7-SB100 plasmid (kindly provided by Z. Izsvak; Addgene #34879; http://n2t.net/addgene:34879; RRID: Addgene_34879) (*87*) at a mass ratio of 19:1 using a Neon Transfection System (Thermo Fisher Scientific) with 3 × 10–ms pulses of 1400 V. Stable bulk cultures were obtained by puromycin selection.

### In vitro cell death and viability assays

#### 3T3-L1 preadipocytes cell death

Cells were treated with 20 μM Z-VAD-fmk (Abcam, ab120382), 5 μM BV6 (Adooq Bioscience, Canada, A14231), 10 μM RIPK1 inhibitor Necrostatin 2 racemate (Nec-1s) (25 mg; SeleckChem, S8641) and costimulated with xFlag-2xStrep-TNF (125 ng/ml) (*22*) for the indicated times.

#### 3T3-L1 adipocytes cell death

After treatments cells were stained with fixable viability dye eFluor 660 eBioscience (Thermo Fisher Scientific, 65-0864-14) 1:1000 in PBS for 10 min, fixed with Fix and Perm kit Foxp3/transcription factor staining (eBioscience, 00-5523-00) for 20 min, filtered in 100-μm strainers, and acquired in FACS Symphony III BD using FACSDiva software (BD) and analyzed in FloJo.v10.8.1. For representative pictures, cells were stained with lipid droplet stain (Linaria, BOT-70065-T) and Sytox Green (Thermo Fisher Scientific, S7020), and images were acquired with S3 Incucyte Live-Cell Analysis (Sartorius).

#### Human and mouse pre- and adipocytes

Viability was determined using CellTiter-Glo assay (G7572, Promega) according to the manufacturer’s instructions. Cells were treated with human TNF-α recombinant protein (PeproTech, 300-01A), doses as indicated in the graphs, and for the M1 TUBE assay, 200 ng/ml was used, and for the inhibitor experiments in adipocytes, the dose was 0.1 ng/ml; protein synthesis inhibitor, CHX (50 μg/ml; Sigma-Aldrich, #239765), 20 μM Z-VAD-fmk (Bachem, #4026865), 500 nM BV6 (Selleck Chemicals, catalog no. S7597), and 10 μM 7-O-Cl-Nec-1 (Sigma-Aldrich, #504297).

### ORO staining

Cells were washed once with PBS before incubation with 4% paraformaldehyde (PFA) at room temperature. After 10 min, PFA was renewed. Samples were stored at 4°C until further analysis. For staining with ORO, cells were washed with 60% isopropanol before addition of ORO staining solution [six parts of ORO stock solution (3.5 g of ORO in 1 liter of isopropanol) were mixed with four parts of distilled water]. After incubation at room temperature for 10 min, the staining solution was removed and cells were washed 3× with distilled H_2_O. Pictures were taken with the Olympus CK40 microscope.

### Western blot

#### Human adipocytes

Cells were lysed in lysis buffer [30 mM tris-HCl (pH 7.4), 150 mM NaCl, 2 mM EDTA, 2 mM KCl, and 10% glycerol] supplemented with 1% Triton X-100, 1× complete EDTA-free protease inhibitor mix (Roche), and 1× phosphatase-inhibitor cocktail 2 (SA). Lysates were denatured in reducing sample buffer before separation by SDS-PAGE (4 to 12% bis-tris; Bolt). Membranes were incubated with primary antibodies at 4°C overnight or for 1 hour at room temperature (RT). Washing of membranes was performed in 1x PBS containing 0.1% Tween 20 (SA) for 3× 10 min before incubation with the horseradish peroxidase (HRP)–conjugated or StarBright Blue 700 fluorescent secondary antibody for 1 hour at RT. Membranes were subsequently imaged on a ChemiDoc Imaging System.

#### Mouse-differentiated adipocytes

Minute Total Protein Extraction Kit for Adipose Tissues/Cultured Adipocytes (AT-022) was used according to the manufacturer’s instructions. For mouse tissues, 100 mg of fat tissue was in 500 μl of radioimmunoprecipitation assay buffer [50 mM tris-HCl (pH 8) and 150 mM NaCl] without detergents and a 5-mm bead to be homogenized in TissueLyser II in two rounds for 2 min at 30 1/s. After tissue disruption detergents (0.5% NP-40 and 0.5% sodium deoxycholate) were added together with EDTA-free protease inhibitor cocktail (Roche) and 1× phosphatase-inhibitor cocktail 2 (Sigma-Aldrich) and deubiquitinase inhibitor (S7130, Selleck Chemicals) sampler were incubated at 4°C for 30 min on agitation. Lysates were denatured with a reduced sample buffer and β-mercaptoethanol at 95°C for 10 min. Proteins were separated by precast 4 to 15% polyacrylamide gels (5678085, Bio-Rad) at 150 V for 50 min, transferred in Trans-Blot Turbo Transfer System (1704150, Bio-Rad) with Transfer Blot mix program in 0.2-μm nitrocellulose membranes (1704158 or 1704159, Bio-Rad), and analyzed by Western blotting. Membranes were blocked for 1 hour RT with tris-buffered saline (TBS) 5% BSA, washed one time with 1× TBS containing 0.1% Tween 20 (SA) before incubation with primary antibodies at 4°C overnight. The next day membranes were washed 3× 5 min with 1× TBS containing 0.1% Tween 20 (9127.1, Carl ROTH) before incubation with the HRP-conjugated for 1 hour at RT. Membranes were subsequently incubated with Clarity Western ECL Substrate (1705060, Bio-Rad) and films (11820, Radiolix). Anti-M1 TUBE (FLAG, UM606, Life Sensors) was performed according to the manufacturer’s instructions.

### Antibodies

#### Human adipocytes

PPARγ (1:1000; rabbit polyclonal, 2443, R&D Systems,) adiponectin (1:1000; rabbit polyclonal, GTX112777, GeneTex), glyceraldehyde-3-phosphate dehydrogenase (1:5000; AbD22549, Bio-Rad), extracellular signal–regulated kinase (ERK) [1:2000; rabbit, 9102, Cell Signaling Technology (CST)], phospho-IκBα [1:1000; immunoglobulin G1 (IgG1), CST, 9246], IκBα (1:1000; rabbit, 9242, CST), phospho-ERK (1:2000; rabbit, 9101, CST), tubulin (dilution 1:5000; AbD22584, Bio-Rad), FLAG [1:1000; IgG1, F3165, Sigma-Aldrich (SA)], HOIP (1:1000; sheep, 68-0013-100, Ubiquigent), SHARPIN (1:2000; rabbit, 14626-1-AP, Proteintech), and HOIL-1 (1:1000; IgG1, clone 2E2, SA). Antibody detecting linear ubiquitin (rabbit) was generated using amino acid sequences as described previously (dilution 1:1000) (*88*).

#### Mouse adipocytes and tissues

The following primary antibodies were used at a 1:1000 dilution: actin (A1978, Sigma-Aldrich), tubulin (T9026, Sigma-Aldrich), RIPK1 (610459, BD), cleaved caspase-8 (9429, Cell Signaling Technology), MLKL (MABC604, Millipore), phospho-IκBα (9246, Cell Signaling Technology), IκBα (9242, Cell Signaling Technology), and phospho-ripk1 (31122S, Cell Signaling Technology), phospho-mlkl (37333, Cell Signaling Technology), and hoip (68-0013-100, Ubiquigent). M1 chains (custom made), cleaved caspase-3 Asp^175^ (9661s, Cell Signaling Technology), perilipin (ab3526, Abcam), phospho-p65 (3033, Cell Signaling Technology),

### Correlation of metabolic syndrome parameters versus LUBAC component expression in VAT

The human data were obtained from the LOBB (www.helmholtz-munich.de/en/hi-mag/cohort/leipzig-obesity-bio-bank-lobb), which includes samples of omental VAT of a cross-sectional cohort. This cohort included 1479 participants, comprising individuals who were either normal weight or overweight (*n* = 31; 52% women; age: 55.8 ± 13.4 years; BMI: 25.7 ± 2.7 kg/m^2^) and those classified as obese (*n* = 1448; 71% women; age: 46.9 ± 11.7 years; BMI: 49.2 ± 8.3 kg/m^2^). The study received approval from the Ethics Committee of the University of Leipzig (approval no: 159-12-21052012) and was conducted in compliance with the Declaration of Helsinki principles. All participants provided written informed consent before their inclusion in the study. VAT samples were obtained during elective laparoscopic abdominal surgeries, following established protocols (*89*, *90*). Body composition and metabolic parameters were assessed using standardized techniques as previously described (*91*, *92*). Participants were excluded on the basis of several criteria: being under 18 years old, having a history of chronic substance or alcohol abuse, smoking within the year leading up to surgery, suffering from acute inflammatory conditions, concurrently using glitazones, having advanced-stage cancers, experiencing a weight loss exceeding 3% in the 3 months before surgery, having uncontrolled thyroid disorders, or being diagnosed with Cushing’s disease. We conducted RNA-seq on ribosomal RNA-depleted samples following the Switching Mechanism at the 5' End of RNA Template (SMARTseq) protocol (*93*). The sequencing was performed as single-end reads using a NovaSeq 6000 (Illumina, San Diego, CA, USA) at the Functional Genomics Center in Zurich, Switzerland. The preprocessing of the data adhered to previously established methods (*94*). In brief, we aligned adapter and quality-trimmed reads to the human reference genome (assembly GRCh38.p13, GENCODE release 32) and quantified gene-level expression with Kallisto version 0.48 (*95*). For samples that had read counts greater than 20 million, we utilized the R package ezRun version 3.14.1 (https://github.com/uzh/ezRun, accessed on 27 April 2023) to downsample them to a maximum of 20 million reads. The normalization of the data was achieved through a weighted trimmed mean (TMM) of the log expression ratios, with adjustments made for age, transcript integrity numbers, and gender—except when analyzing male and female samples separately. All analyses were carried out using R version 4.3.1 (www.R-project.org).

### RNA isolation and qRT-PCR

#### Human adipocytes

RNA was isolated with the Quick-RNA Miniprep Kit (Zymo) according to the manufacturer’s instructions. cDNA was synthesized using SuperScript II Reverse Transcriptase (Thermo Fisher Scientific) with random primers (Thermo Fisher Scientific). Quantitative reverse transcription polymerase chain reaction (qRT-PCR) was performed using SsoAdvanced Universal SYBR Green Supermix (Bio-Rad) on a Bio-Rad CFX Connect Real-Time PCR Detection System with the following protocol: 95°C for 30 s, and then 40 cycles of 95°C for 5 s, followed by 60°C for 30 s. Treatment times and concentrations are indicated in figures and figures legends.

#### Mouse adipose tissue and liver

GWAT samples were processed according to the manufacturer’s instructions using the RNeasy Lipid Mini Kit (74804, QIAGEN). For tissue disruption, 100 mg of fat tissue were resuspended in 1 ml of QIAyol Lysis buffer (RLT Plus + DX) homogenized in TissueLyser II using a 5-mm bead in in two rounds of 2 min at 30 1/s. For liver, samples were processed according to the manufacturer’s instructions using the RNeasy Mini kit (QIAGEN, 75142). For tissue disruption, 100 mg of liver was resuspended in 2 ml of lysis buffer RLT plus with β-mercaptoethanol (10 μl of β-ME were added to every 1 ml of RLT buffer), and samples were homogenized in TissueLyser II using a 5-mm bead in in two rounds for 2 min at 30 1/s. For both tissues, a deoxyribonuclease (DNase) digest kit was used, involving the addition of 350 μl of buffer RW1 to the RNeasy Mini spin column, followed by the addition of DNase I. The resulting RNA was evaluated for purity and concentration using a NanoDrop spectrophotometer from Tecan Reader.

### RNA-seq from GWAT and liver

For RNA-seq, we used 2 μg of total RNA per sample concentration range 50 to 200 ng/μl, at least 10-μl volume, checking the quality by RNA integrity number > 7, OD_260/280_ = 1.8 to 2.1 and OD_260/230_ > 1.5. mRNA libraries were prepared by mRNA Seq (polyadenylated selection) using External RNA Controls Consortium (ERCC) RNA Spike-In controls. Libraries were prepared using the Illumina Stranded TruSeq RNA sample preparation kit. ERCC RNA Spike-In Mix (Thermo Fisher Scientific) was added to the samples before library preparation. Library preparation started with 1000 ng of total RNA. After polyadenylate selection (using poly-T oligo-attached magnetic beads), mRNA was purified and fragmented using divalent cations under elevated temperature. The RNA fragments underwent RT using random primers. This was followed by second strand cDNA synthesis with DNA polymerase I and ribonuclease H. After end repair and A-tailing, indexing adapters were ligated. The products were then purified and amplified (15 cycles) to create the final cDNA libraries. After library validation and quantification (Agilent TapeStation), equimolar amounts of the library were pooled. The pool was quantified by using the Peqlab KAPA Library Quantification Kit and the Applied Biosystems 7900HT Sequence Detection System. The pool was sequenced on an Illumina NovaSeq6000 sequencing instrument with a PE100 protocol aiming for 40 million clusters per sample.

For GWAT, RNA-seq dataset was processed on the CHEOPS HPC cluster at the University of Cologne using the nf-core (*96*) RNA-seq pipeline (v3.7) (*97*) within a Singularity environment (https://nf-co.re/rnaseq/3.19.0/) and managed by Nextflow (v21.10.6) (*98*). Quality control, including adapter trimming and sequence filtering, was performed with Trim Galore (v0.6.7) (www.bioinformatics.babraham.ac.uk/projects/trim_galore/), ensuring high-quality reads. Read quality was assessed via MultiQC (v1.11) (*99*), aggregating FastQC (www.bioinformatics.babraham.ac.uk/projects/fastqc/), Picard (https://broadinstitute.github.io/picard/), and Preseq (*100*) reports. No issues were detected. Reads were aligned to the GRCh38 reference genome with STAR (v2.7.10a) (*101*), and gene quantification was done using Salmon (v1.5.2) (*101*). Differential expression analysis was performed in DESeq2 (v1.38.1) (*102*), with multiple testing corrections applied using the Benjamini-Hochberg method. GO analysis, focusing on genes with an absolute log_2_ fold change > 1 and adjusted *P* value < 0.05, was carried out using gprofiler2 (v0.2.1) (*103*). Positively and negatively enriched pathways were identified separately using default parameters, including g_SCS correction. Pearson’s correlation tests were initially performed on the log_2_ fold changes of all genes between the Ctrl (HFD versus CD) and CD (*Hoip^A-KO^* versus Ctrl) comparisons. Genes were then grouped by significance in either or both comparisons, and correlation analyses were repeated for each group. All tests showed strong to very strong correlations (0.77 to 0.94), with statistically significant results. Cell-type deconvolution was performed using the deconvolute function from Granulator (v1.6.0) and the dtangle method (*104*). The patterns of each line indicate how each model predicts changes in cell proportions under the different conditions. Hence, the overlapping lines indicates that the models predict similar changes in cell proportions. As input, we used Salmon’s transcripts per million (TPM) values generated by the nf-core pipeline and perigonadal samples from the single-nuclei dataset GSE176171. The preprocessed Seurat object (mouse_all.rds) was sourced from https://gitlab.com/rosen-lab/white-adipose-atlas and restricted to male perigonadal tissue. GSEA was performed in R (v4.3.0) using the clusterProfiler (v4.10.1) (*105*) package. Gene sets for apoptosis, necroptosis, ferroptosis, and adipokines were obtained from Kyoto Encyclopedia of Genes and Genome0 via KEGGREST (https://github.com/Bioconductor/KEGGREST) (v1.42.0). Pyroptosis (GO:0070269), NF-κB, and MAPK gene sets were sourced from the Molecular Signatures Database (MSigDB) using msigdbr (v10.0.1) (*106*–*108*, *109*). Genes were ranked by score resulting from multiplying –log_10_(*P* value) with the sign of their fold change. For each contrast, the ranked gene lists and the aforementioned gene sets were used as input for GSEA (10,000 permutations; minimum/maximum gene set size: 5/1000; no initial *P* value cutoff). Enrichment plots for selected pathways were generated with enrich plot (https://bioconductor.org/packages/release/bioc/html/enrichplot.html) (v1.22.0). All analyses used the fixed random seed 123 for reproducibility. GSEA performs a permutation test and reports *P* values adjusted using the Benjamini-Hochberg procedures as well as *q* values, which estimate the minimum false discovery rate at which a gene set is considered significant.

For liver samples, alignment of the RNA-seq data was performed using the Nextflow RNA-seq pipeline (*110*) (version 3.13.2) using default options. The reads were mapped to the mouse genome (NCBI RefSeq assembly GCF_000001635.27). The count data were analyzed using the Bioconductor package DESeq2 (*111*) (version 1.46.0) in R (*112*) (version 4.4.2) and normalized using the size factors derived from the median of ratios. Two different negative binomial generalized linear models were used for differential gene expression analysis: ~ genotype + diet for estimation of diet or genotype effect and ~ “genotype_diet” for between group comparisons. Log_2_ fold Changes and *P* values were obtained using a Wald test with Benjamin-Hochberg correction for multiple testing. An established 25-gene signature was used as a marker for non-alcoholic fatty liver disease (NAFLD) (*49*). Plots were created using the R package ggplot2 (*113*) (version 3.5.1)

### Histological assessment and quantifications

At the indicated end points, tissues were harvested and fixed overnight in a 10% neutral buffered formalin solution (Sigma-Aldrich, HT501128-4L) and paraffin-embedded. Tissue sections (4 μm) were processed as described. Colon and small intestine were isolated from the mouse, longitudinally cut, and washed with PBS to remove feces. Intestinal tissues were rolled up from proximal to distal and fixed in 4% PFA overnight at 4°C. Afterward, sections were paraffin-embedded and cut to 4-μm thickness. Paraffin sections were stained with H&E and acquired in the Slide Scanner (Hamamatsu S360) at 20×. For intestine, the histological score formalin-fixed and paraffin-embedded intestinal Swiss rolls were sectioned (3 μm) and stained with H&E.

#### Adipocyte distribution

The whole scan tissue of six mice per group was selected from 20× H&E scanned sections of GWAT or SWAT. Three mice of two different cohorts were selected, being in CD and HFD littermates’ controls in both cases the dimensions of the image were 4992 pixels by 2640 pixels for mice with 16 weeks of diet and 9984 pixels by 5280 pixels for 12-week-old CHOW diet mice. The images were analyzed with a modified version of the Adiposoft plugin (Fiji, v1.54). Contrast enhancement was removed in this modified version. Instead of the global thresholding using the percentile method, autolocal thresholding was performed in a 15-pixel radius using the mean pixel value. The prominence of local maxima in the distance map for water shedding was set to 7. Particles were identified as valid adipocytes if their diameter fell into the range between 10 and 200 μm and their circularity was between 0.27 and 1.00. The whole data obtained were used to generate distribution plots. Individual averages of adipocyte numbers and area were used to generate bar plots.

#### Liver steatosis

H&E staining of 16-week diet and 18-month-old mice livers were evaluated by an experienced clinical pathologist who was blinded for mouse ID, age, genotype, or diet. Standardized scores for Nonalcoholic Fatty Liver Disease (NAFLD) were performed according to the NAFLD Activity Score (NAS): Steatosis (0 to 3), lobular inflammation (0 to 2), hepatocellular ballooning (0 to 2), and fibrosis (0 to 4) (*114*).

#### Intestinal histological evaluation

The scoring system used is described in (*70*). In brief, histopathology scores comprise four parameters: epithelial hyperplasia, epithelial injury, tissue inflammation, and epithelial cell death. Histological subscores for each parameter are as follows: 0, absent; 1, mild; 2, moderate; 3, severe. An “area factor” for the fraction of affected tissue was assigned and multiplied with the respective parameter score (1 = 0 to 25%; 2 = 25 to 50%; 3 = 50 to 75%; 4 = 75 to 100%). Each area was scored individually and multiplied with the correlating area factor. Total histology score was calculated as a sum of all parameter scores multiplied by their area factors. The maximum score was 48. The evaluation was performed in a blinded fashion.

### Immunofluorescence

#### D163, Mac2, and perilipin

Samples were deparaffinized using xylene every 5 min and alcohol series every 3 min. Citrate Ph6 was used for Antigen Retriever. Samples were washed five times for 5 min in PBS–0.3% Triton X-100. Samples were blocked using 1% BSA (P6154-100gr, Biowest) in PBS–0.3% Triton X-100 for 1 hour. Samples were incubated overnight with primary antibody (Ab) (see list). Afterward, samples were washed five times for 5 min 0.3% Triton–PBS (the last two times in cuvette) and incubated for 1 hour at RT with secondary Ab (see list). Followed by four washes for 5 min, 0.3% Triton-PBS in cuvette, DAPI staining was performed for 10 min RT. TrueBlack (23007, Biotium) (1:20 diluted in ethanol) was added for 15 s, followed by two washes of 5 min in PBS and two washes of distilled water in a cuvette. DAKO Fluorescence Mounting Medium (S3023, DAKO) was used to seal the samples. Primary antibodies were diluted in Ab in 1% BSA and 0.3% Triton-PBS in the following concentrations: anti-Mac2 (rat) (1:1000; CL8942AP, Cedarlane), anti-CD163 (rabbit) (1:100; ab182422, Abcam), and anti-PerilipinA (goat) (1:200; ab61682, Abcam) and secondary Abs in the following concentrations: donkey anti-rat DyLight488 (1:200; SA5-10026, Invitrogen), donkey–anti-rabbit Cy3 (1:200; 711-165-152, the Jackson Laboratory), donkey-anti-goat AF647 (1:200; A 21447, Invitrogen), and DAPI (1:10,000; D1306, Invitrogen). Images were taken with the Olympus BX53 epifluorescence microscope at 10× for quantification and 20× or 40× for the figures and quantified using cellSens software.

#### TUNEL staining

Perigonadal adipose tissue (GWAT) sections were used for TUNEL staining (Promega, G3250) according to the manufacturer’s specifications and DAPI to identify the nucleus structure. TUNEL-positive cells were determined by using DNase I as specified in the kit. Negative control is without enzyme. Images were taken with the EVOS scanner at 20× and quantified using a positive cell detection tool on QuPhat 5.0 with a DAPI threshold of 25 and a cell expansion mask of 25 μm. Positive threshold was determined in the positive control. The number of positive nuclei was normalized to the total nuclei and total tissue area of each corresponding section; visible blood vessel areas were excluded from the analysis due to autofluorescence.

#### Cleaved Caspase-3 staining

The slides were then incubated in 1× citrate buffer (pH 6.0; 10X stock, catalog no. C999) for 10 min before heat-induced antigen retrieval. Slides were simmered in a microwave for 7 min. Afterward, they were cooled to RT. Tissue sections were permeabilized by incubating them in 0.2% Triton X-100 (Sigma-Aldrich, catalog no. T8787), prepared in animal-free blocker (catalog no. SP-5035-100), for 10 min, and slides were washed with 1× TBS for 5 min and then with 1× Tris-Buffered Saline with 0.1% (v/v) Tween-20 (TBS-T) for 5 min. Next, the slides were incubated with blocking solution (catalog no. VEC-SP-6000) for 10 min and then with animal-free blocker for 1 hour. After blocking, tissues were incubated overnight at 4°C with Cleaved Caspase-3 (Asp^175^) antibody (catalog no. 9661S; 1:50 dilution in animal-free blocker). The following day, the slides were washed three times for 10 min each with 1× TBS-T and then incubated for 1 hour with goat anti-rabbit IgG (H + L) cross-adsorbed secondary antibody and Alexa Fluor 568 (catalog no. A11011; 1:300 dilution in 1× TBS-T). Slides were then washed six times for 10 min each with 1× TBS-T. Last, the slides were incubated with DAPI (1:1000) for 10 min, rinsed in 1× TBS-T, and mounted using Molecular Probes ProLong Gold Antifade Mountant (catalog no. P10144). EVOS scanner at 20× and quantified using positive cell detection tool on QuPhat 5.0 with a DAPI threshold of 25 and a cell expansion mask of 25 μm. Positive threshold was determined by negative control without primary Ab. The number of positive nuclei was normalized to the total nuclei and total tissue area of each corresponding section; visible blood vessel areas were excluded from the analysis due to autofluorescence.

### Immunohistochemistry

For insulin (1:6400 diluted in animal-free blocker, C27C9, Cell Signaling Technology), CD45 (1:100; 14-0451-82, Thermo Fisher Scientific), and F4/80 (1:100; MCA497R, Bio-Rad), antigen retrieval was done by using citrate buffer (pH 6; Sigma-Aldrich, C9999) in pancreas or GWAT sections (5 μM) by simmering in microwave for 5, 10, 10, and 7 min, respectively. The sections were incubated with BLOXALL Endogenous Blocking Solution (VEC-SP-6000, Vector Laboratories) to block endogenous peroxidases. This is followed by 30-min incubation with animal-free blocker (VEC-SP-5035, Vector Laboratories). Following overnight incubation at 4°C, with primary antibodies, sections were washed with TBS-T (pH 7.6, 0.5% Tween) three times for 10 min. Sections were incubated with the immPRESS HRP Goat Anti-Rabbit IgG Polymer Detection Kit and peroxidase (MP-7451, Vector Laboratories) for 30 min. Following three times for 10 min washes, the color was developed by using HIGHDEF DAB Chromogen/Substrate Set (ENZ-ACC105-0200, Enzo). The slides were scanned by slide scanner (Hamamatsu S360). The positive area was calculated by using QuPath 0.4.0.

### Intravital imaging

Functional intravital imaging was performed using a two-photon microscope (LSM MP7; Zeiss, Germany), as previously described (*115*). Approximately 45 min before recording, the mice received bolus tail vein injections of TMRE (1.5 mg/kg; mitochondrial membrane potential marker), Hoechst 33258 (15 mg/kg; nuclear marker), PI (3 mg/kg; cell death marker), and fluorescein-coupled dextran 10 kDa (4 mg/kg; to visualize blood flow); all dyes were purchased from Thermo Fisher Scientific. To image the epididymal fat, the mice were anesthetized with an intraperitoneal injection of a mixture of 2% rompun (20 mg/kg) and ketamine (120 mg/kg); after loss of all reflexes, a small midline incision was made in the abdominal wall, the mouse was placed on a lateral position, and the epididymal fat was exposed and placed on a coverslip fixed on an image platform. The exposed adipose tissue was covered with saline-soaked gauze to avoid dryness. The mice were kept on maintenance isoflurane anesthesia during the recording. At least three mice were recorded from each condition.

### Liver lipidomics

Glycerophospholipid [phosphatidylcholine (PC), phosphatidylethanolamine (PE), phosphatidylinositol (PI), phosphatidylserine (PS), phosphatidylglycerol (PG), phosphatidic acid (PA)], DAG, triacylglycerol (TAG), and CE species in mouse liver were analyzed by Nano-Electrospray Ionization Tandem Mass Spectrometry (Nano-ESI-MS/MS) with direct infusion of the lipid extract (Shotgun Lipidomics) using a TriVersa NanoMate infusion system operated by the ChipSoft 8.3.1 software (Advion) and a QTRAP 6500 mass spectrometer operated by the Analyst 1.7.2 software (SCIEX). Samples of mouse liver tissue were homogenized in Milli-Q water (1 mg/10 μl) using the Precellys 24 Homogenisator (Peqlab) at 6500 rpm for 30 s. The protein content of the homogenate was routinely determined using bicinchoninic acid. For the analysis of Glycerolipids (GPL) species, 15 μl of the homogenate was used for lipid extraction, whereas 20 μl was used for the analysis of DAG and 5 μl for TAG and CE species. The homogenate aliquots were brought to a volume of 500 μl with Milli-Q water. For the extraction of GPL species, 1.875 ml of methanol/chloroform [2:1 (v/v)] and internal standards from Avanti Polar Lipids [122 pmol of PC (17:0–20:4, LM1002), 138 pmol of PE (17:0 to 20:4; LM1102), 111 pmol of PI (17:0 to 20:4, LM1502), 118 pmol of PS (17:0 to 20:4, LM1302), 60 pmol of PG (17:0 to 20:4, LM1202), 72 pmol of PA (17:0 to 20:4, LM1402)] were added. DAG, TAG, and CE species were extracted with 1.875 ml of chloroform/methanol/37% hydrochloric acid [5:10:0.15 (v/v/v)] and 20 μl each of 4 μM d5-DAG Internal Standard Mixtures I and II (LM6001 and LM6004, Avanti Polar Lipids) or 30 μl of 4 μM d5-TAG Internal Standard Mixture I (LM6000, Avanti Polar Lipids) and 2.28 nmol of cholesteryl ester 19:0 (LM4000, Avanti Polar Lipids). Lipid extraction Nano-ESI-MS/MS analysis were performed as previously described (*116*). Mass spectra were processed by the LipidView 1.2 software (SCIEX) for identification and quantification of lipids. Endogenous lipid species were quantified by referring their peak areas to those of the respective internal standard. The calculated lipid amounts were normalized to the protein content of the tissue homogenate. For the representation of TAG species in heatmaps (fig. S4D), the prefix must be read as the first fatty acid containing the specified number of carbons, while the last two fatty acids are represented as the sum of both species and their double bonds, e.g., “18:3-xx:y” means that the first fatty acid has 18 carbon atoms and three double bonds. Subsequently, “xx” represents the sum of carbon atoms, and “y” the sum of double bonds of the other two fatty acids.

### Echocardiography

Transthoracic echocardiography was performed in isoflurane-anesthetized mice [Isoflurane-Piramal, Piramal Critical Care, Voorschoten, The Netherlands; 5% (v/v) for induction and 2% (v/v) for maintenance of anesthesia]. Parasternal long axis (PSLAX) and parasternal short axis of the heart were imaged in B-Mode, M-Mode, and electrocardiogram-triggered kilohertz (EKV) visualization using a Vevo 3100 ultrasound system (FUJIFILM VisualSonics, Toronto, ON, Canada) equipped with a MX550D transducer (25 to 55 MHz, center transmit: 40 MHz, axial resolution: 40 μm) by a blinded examiner. Measurements were analyzed using Vevo LAB v5 (VisualSonics, FUJIFILM, Tokyo, Japan) by two blinded investigators. Left ventricular ejection fraction (LV-EF) and cardiac output were determined by planimetry of end-systolic [LV end-systolic volume (LVESV) and end-diastolic [LV end-diastolic volume (LVEDV) LV volumes in the B-mode/EKV PSLAX view.
