# Supplementary material for: Linear ubiquitination prevents lipodystrophy and obesity-associated metabolic syndrome
Source: Sci Adv. 2025 Sep 17;11(38):eadw2539. doi: 10.1126/sciadv.adw2539 (PMC12442851; doi:10.1126/sciadv.adw2539)
Supplement: Supplementary file 1 — Figs. S1 to S11 Legends for movies S1 and S2 Legends for tables S1 to S5 [file sciadv.adw2539_sm.pdf]

Science Advances
AAAS

Supplementary Materials for

Linear ubiquitination prevents lipodystrophy and obesity-associated metabolic syndrome

Ximena Hildebrandt *et al.*

Corresponding author: Nieves Peltzer, n.peltzer@ibmg.uni-stuttgart.de

Sci. Adv. **11**, eadw2539 (2025)
DOI: 10.1126/sciadv.adw2539

The PDF file includes:

Figs. S1 to S11
Legends for movies S1 and S2
Legends for tables S1 to S5

Other Supplementary Material for this manuscript includes the following:

Movies S1 and S2
Tables S1 to S5

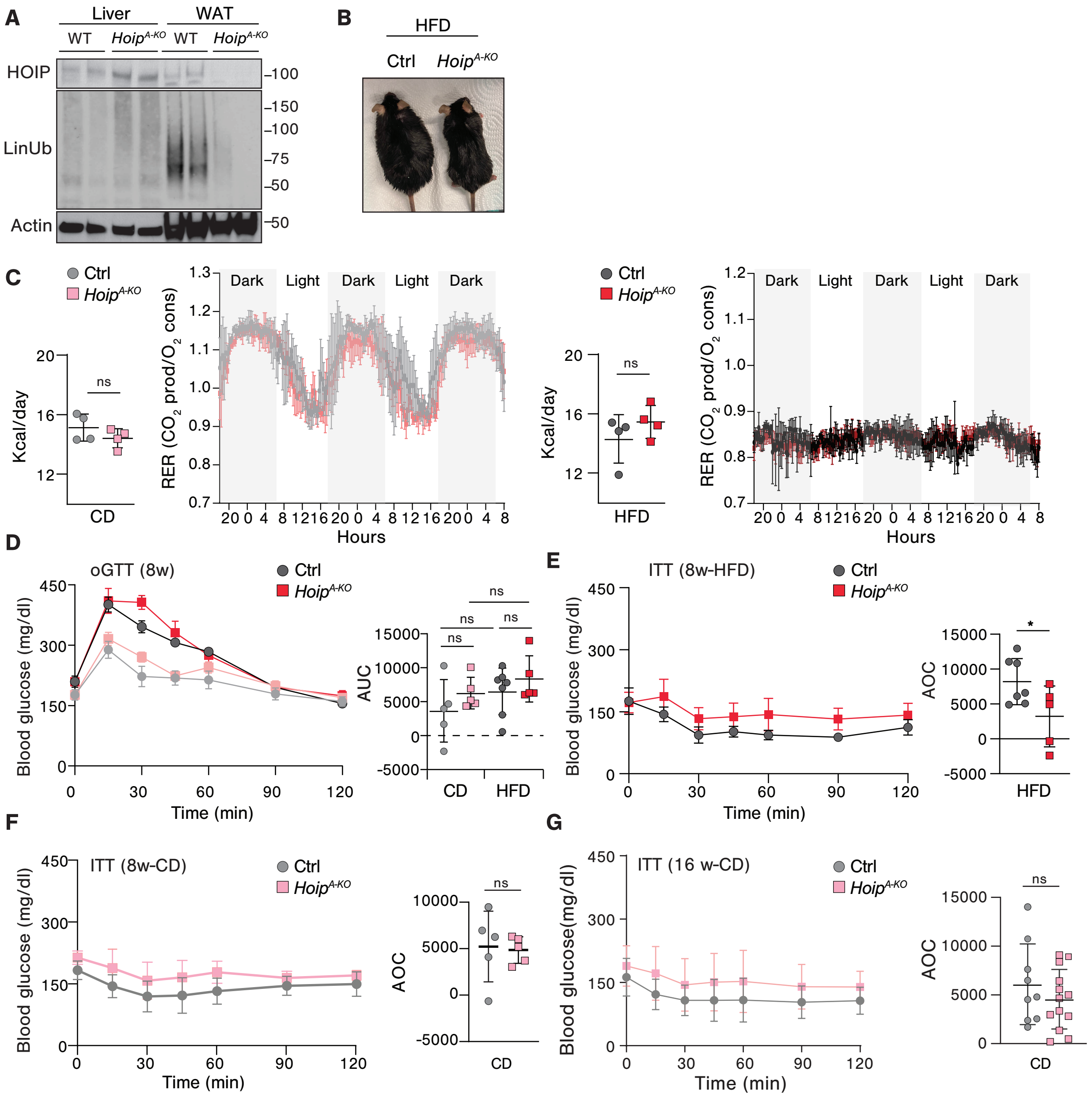

Supplementary Figure 1. Impaired linear ubiquitination in adipocytes accelerates insulin resistance onset upon HFD. Related to Figure 1

(A) Western blot of ScWAT protein lysates from 12-week-old mice. (B) Representative picture of 16-week-HFD-fed mice. (C) Calorimetry and Respiratory exchange ratio (RER) of mice at 15 weeks of diet measured in metabolic cages (n=4). (D) Oral Glucose Tolerance Test at 8 weeks of diet (n=5-7). (E, F) Insulin Tolerance Test at 8 weeks of diet (n=5-7).

(G) Insulin Tolerance Test at 16 weeks of CD diet, (n=9-13). All values represent the mean +/- SD. Statistics: C, E, F, G: T-test: p-values: (**) p<0,01, (*) p<0,05, (ns) not significant; D> 1-way ANOVA of selected comparison with Bonferroni correction, p-values GP: (#) p<0,0001, (***), p<0,0002;(**) p<0,0021; (*) p<0,0332; (ns) p->0,0333.

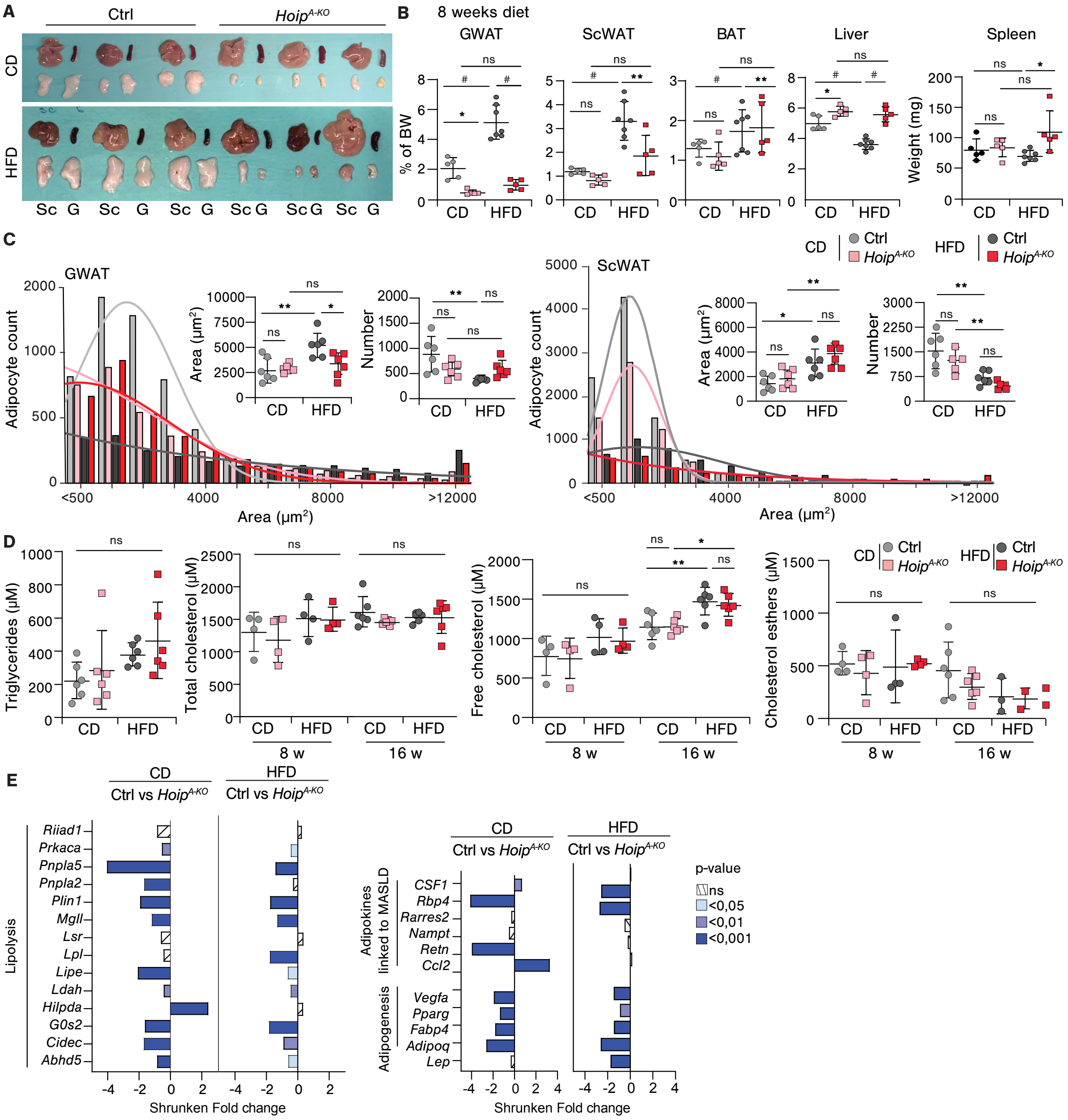

Supplementary Figure 2. Adipocyte-specific HOIP deficiency causes lipodystrophy but not dyslipidemia or systemic inflammation. **R**elated to Figure 2.

(A) Representative images of the indicated tissues after 16 weeks on diet (Sc: subcutaneous white adipose tissue, G: gonadal white adipose tissue). (B) Tissue weight normalized to total body weight after 8 weeks on diet. (C) Distribution of adipocyte area in GWAT and ScWAT after 16 weeks on diet, quantified using Adipo-Fiji software. Data include mean adipocyte area and number of individual mice (n: 5–6). (D) Serum levels of triglycerides, total and free cholesterol, and cholesterol esters after 8 weeks (n: 4) or 16 weeks (n: 6) on diet, as indicated. Missing values were below the detection limit. (E) Shrunken fold change of selected genes involved in lipolysis, adipokine signaling, MASLD, and adipogenesis from bulk RNA-seq analysis of GWAT after 16 weeks on diet (n = 2–3 pools of 3 mice per group), normalized to WT control. All values represent mean ± SD. Statistics: For panels B, C, and D, one-way ANOVA grouped by diet with Bonferroni correction for multiple comparisons: (#) p < 0.0001; () p < 0.0002; () p < 0.0021; () p < 0.0332; (ns) p > 0.0333. For panel E, multiple testing correction was performed using DESeq2 with the Benjamini-Hochberg method; p-values are indicated in the legend.

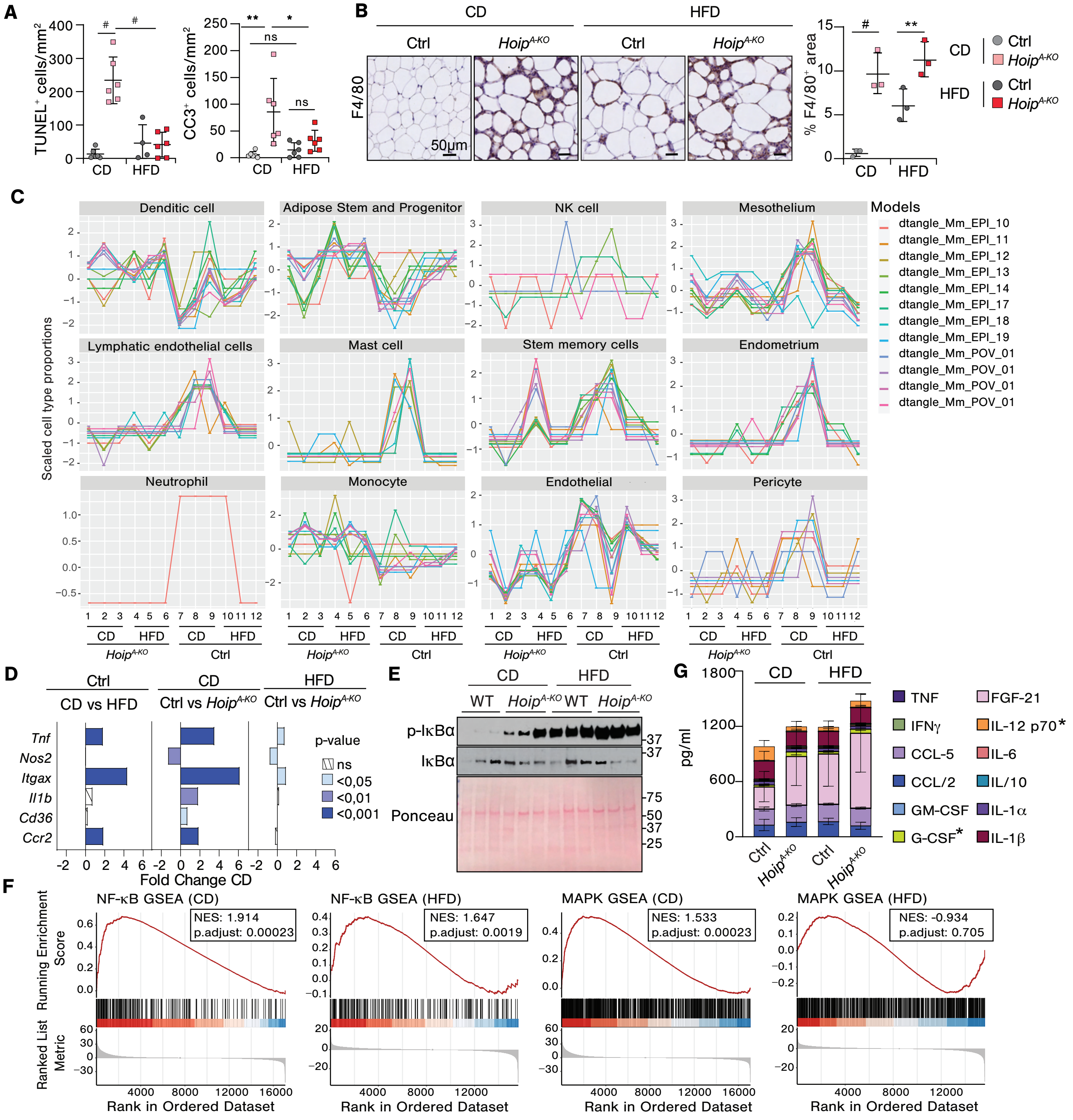

Supplementary Figure 3. Adipocyte-specific HOIP deficiency causes adipocyte death and macrophage infiltration. Related to Figure 2. (A) Quantification of immunostainings from Fig. 2F (TUNEL and cleaved caspase 3) (n: 5-6). (B) Representative images (left) and quantification (right) of F4/80 immunohistochemistry in GWAT after 16 weeks of diet (n:3). (C) Deconvolution of bulk RNA-seq data from GWAT after 16 weeks of diet (n:2–3 pools of 3 mice) using cell type–specific gene signatures for the indicated cell types. (D) Shrunken fold change of selected pro-inflammatory genes from bulk RNA-seq analysis of GWAT after 16 weeks of diet (n:2–3 pools of 3 mice per group). (E) Western blot analysis of ScWAT protein lysates after 16 weeks of diet. (F) Gene Set Enrichment Analysis (GSEA) from bulk RNA-seq of GWAT after 16 weeks of diet (n:2–3 pools of 3 mice per group). NES: normalized enrichment score; p.adjust: adjusted p-value (G) Cumulative cytokine concentrations in serum after 16 weeks of diet, determined by multiplex assay (n:8; values below detection limit excluded). Values represent mean ± SD. Statistics: For panels A, B, and G, one-way ANOVA of selected groups with Bonferroni correction: (#) p<0.0001; () p<0.0002; () p<0.0021; () p<0.0332; (ns) p>0.0333. For panel D, multiple comparisons were corrected using the Benjamini-Hochberg method, including g_scs correction and Pearson correlation tests on log2 fold change. For panel F, GSEA performs a permutation test and reports p-values adjusted using the Benjamini-Hochberg procedure as well as q-values.

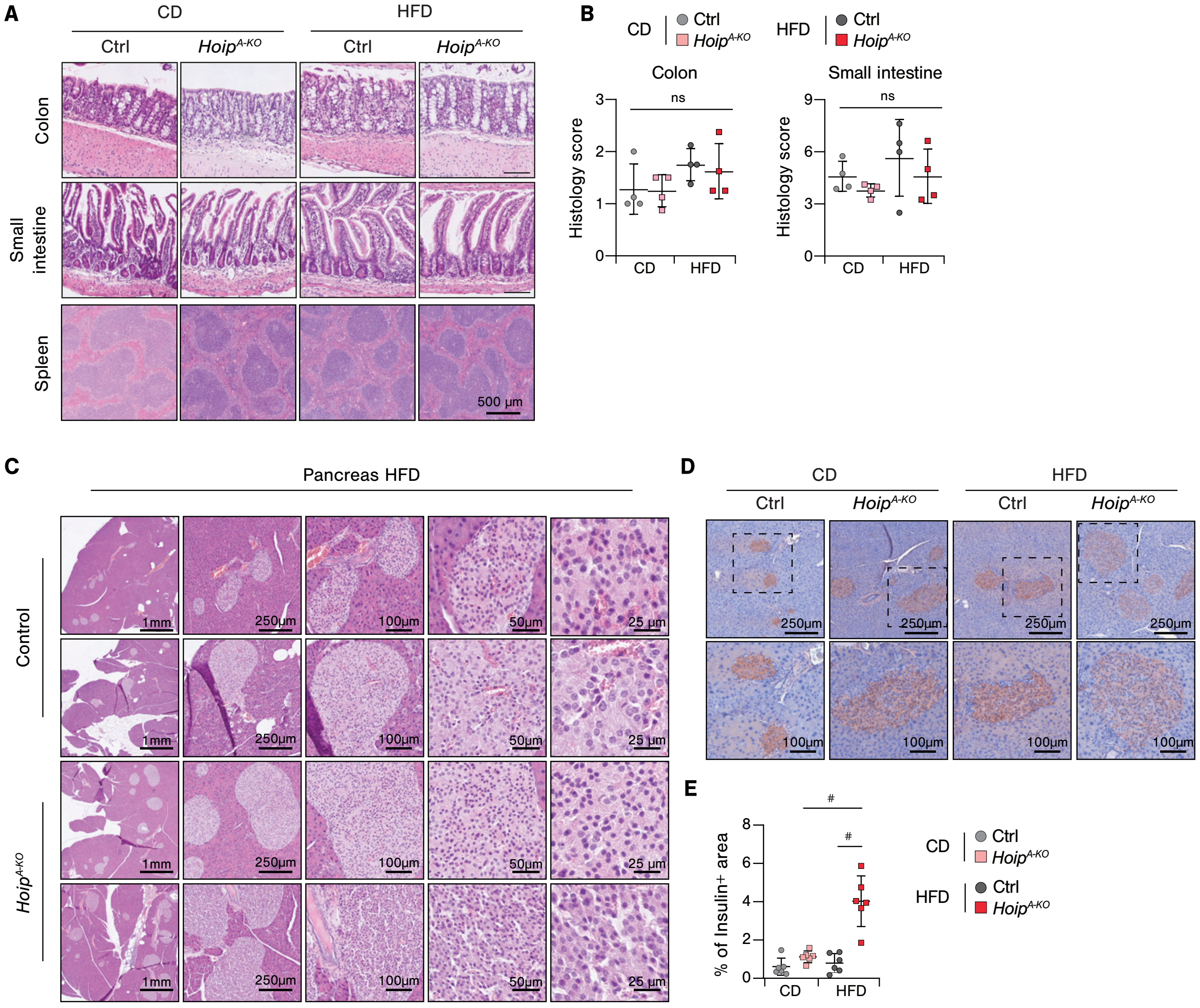

Supplementary Figure 4. Loss of LinUb in adipocytes exacerbates pancreatic islet hyperplasia during obesity. Related to Figure 3
(A) Representative H&E images (Swiss rolls) of different intestinal sections (jejunum, ileum, duodenum, and colon) and spleen from mice after 16 weeks of diet. (B) Histological scoring of the intestinal sections shown in (A) (n:4). (C) Close-up representative H&E images of the pancreas from HFD-fed mice. (D) Insulin immunostaining of the pancreas after 16 weeks of diet. (E) Quantification of islet area (Insulin+) normalized to the total area of the pancreas (n: 6). All values represent mean ± SD. Statistics: One-way ANOVA for selected groups with Bonferroni correction for multiple comparisons. p-values GP: (#) p<0.0001; () p<0.0002; () p<0.0021; () p<0.0332; (ns) p>0.0333.

A

	Steatosis	Lobullar Inflammation	Balloning
Ctrl CD vs Ctrl HFD	*	ns	**
Ctrl CD vs *Hoip*$^{A\text{-}KO}$ CD	#	ns	#
Ctrl HFD vs *Hoip*$^{A\text{-}KO}$ HFD	ns	ns	ns
Hoip$^{A\text{-}KO}$ CD vs *Hoip*$^{A\text{-}KO}$ HFD	#	*	*

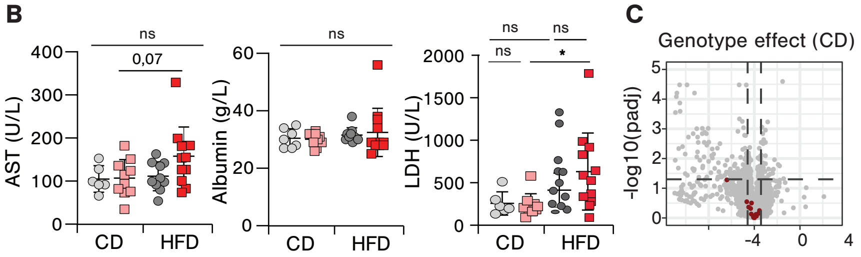

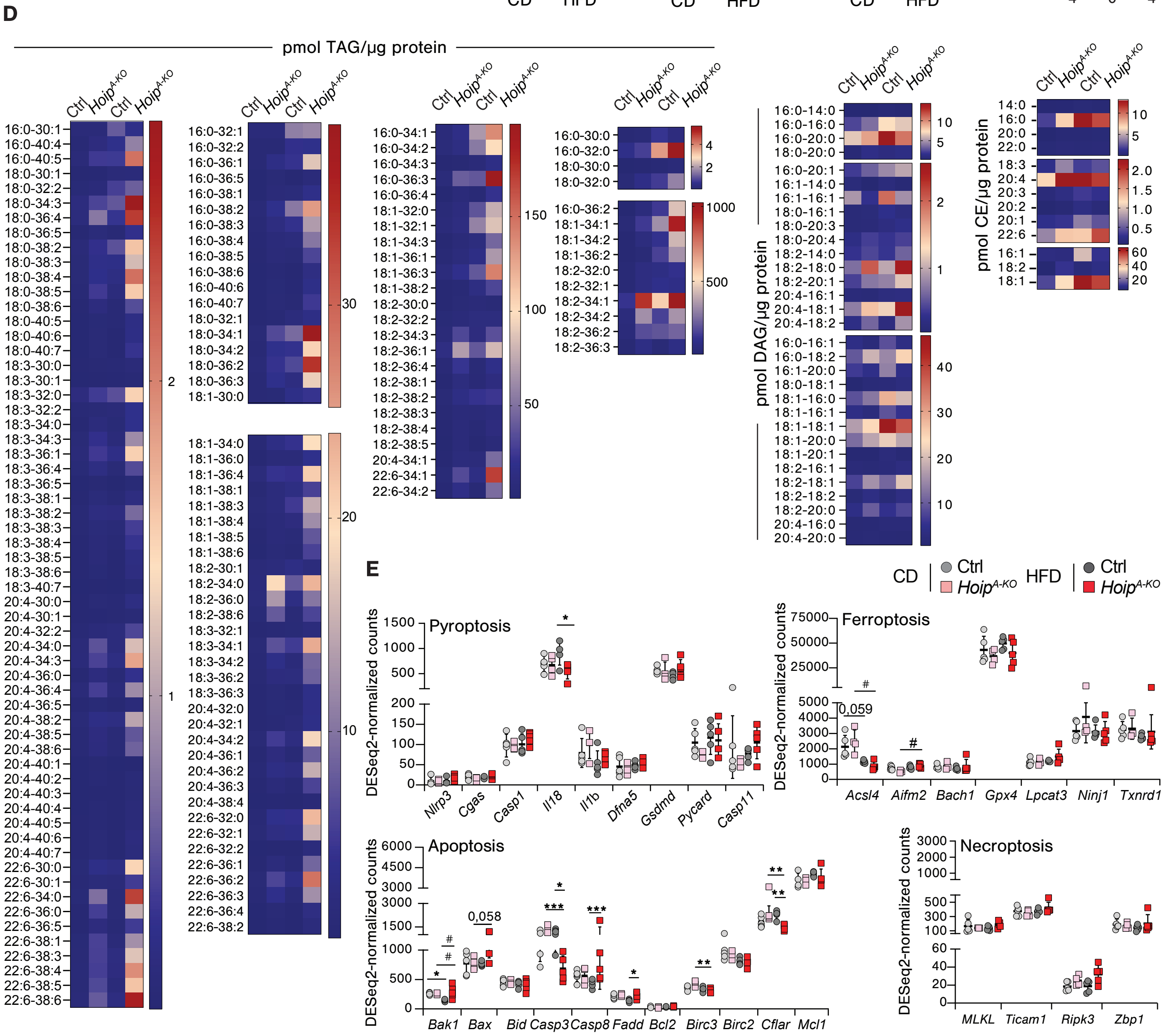

Supplementary Figure 5. Loss of LinUb in adipocytes predisposes mice to MASLD during obesity. Related to Figure 3.
(A) Liver steatosis scores from Fig.3 A-B, with corresponding statistics split by category (n: 14–17). (B) Serum levels of AST, albumin, and LDH after 16 weeks of diet (n: 6–12). (C) Left: Volcano plot of liver RNA-seq data comparing selected NASH signature genes between *Hoip*$^{A\text{-}KO}$ and control mice on a control diet (CD). The x-axis shows $\log_2$ fold change (*Hoip*$^{A\text{-}KO}$/WT); the y-axis shows $-\log_{10}$(adjusted p-value) (padj). (D) Heatmaps of liver lipidomics after 16 weeks of diet (n: 5). (E) DESeq2-normalized counts from liver RNA-seq of selected cell death–related genes (n: 5). All values represent mean ± SD. Statistics: For A and B, one-way ANOVA for selected groups with Bonferroni correction for multiple comparisons. p-values GP: (#) p<0.0001; () p<0.0002; () p<0.0021; () p<0.0332; (ns) p>0.0333. For C and E, $\log_2$ fold changes and p-values were obtained using a Wald test with Benjamini-Hochberg correction for multiple testing. p-values: (**) p<0.01, (*) p<0.05, (ns) not significant.

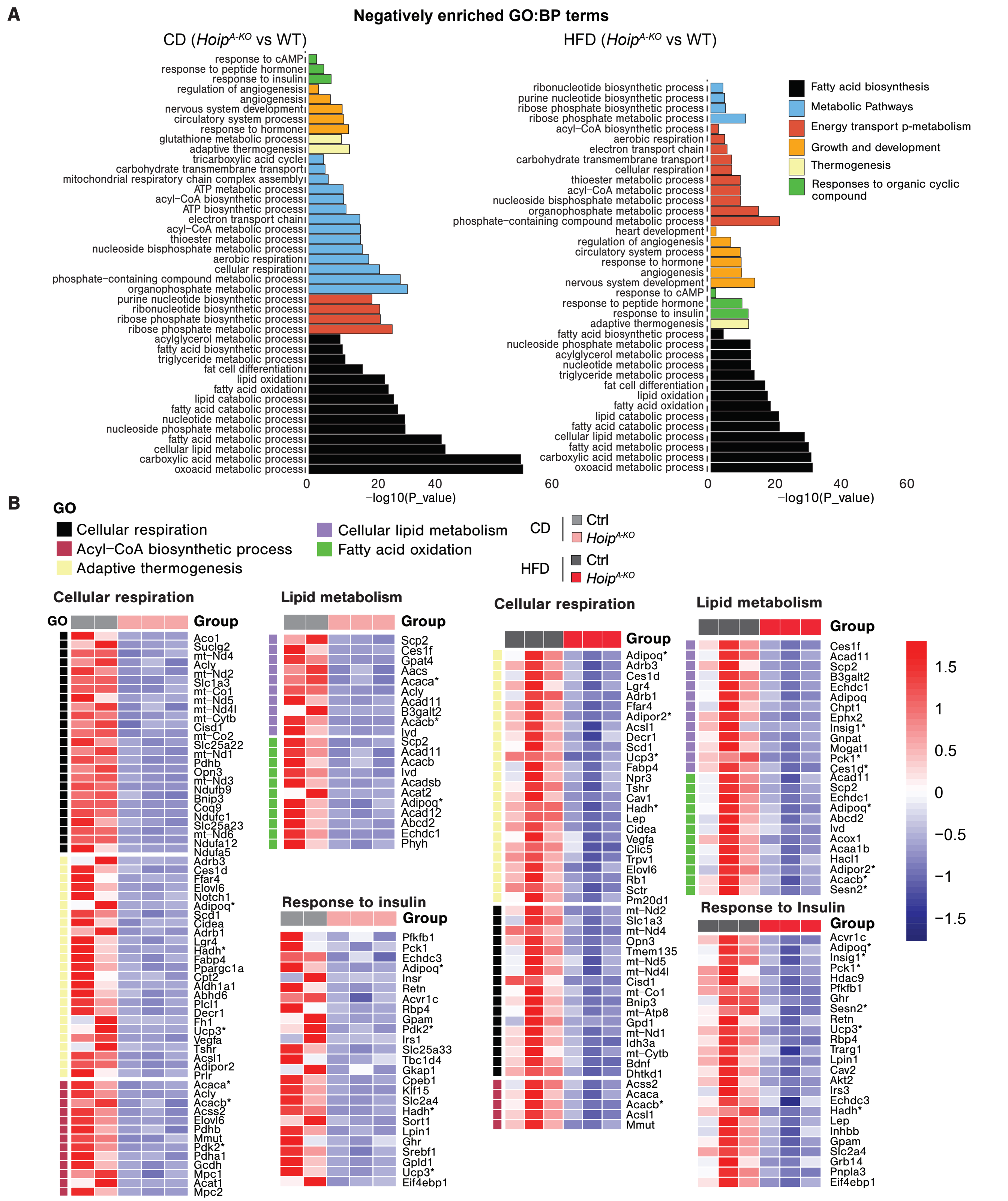

Supplementary Figure 6: HOIP loss leads to a downregulation of genes involved in several metabolic pathways. Related to Figure 4
(A) Bulk RNAseq analysis of GWAT after 16-weeks-diet. (n=2-3 pools of 3 mice). Negatively enriched GO terms in CD HoipA-KO vs WT (left) and HFD HoipA-KO vs wt (right).
(B) Heatmaps of selected pathways from downregulated genes in *Hoip*$^{A\text{-}KO}$ mice. *indicates repeated genes
Statistics: For DEA multiple comparisons were corrected using Benjamini-Hochberg. For GOs adjusted were p-value <0.05. Enriched pathways were identified separately using default parameters, including g_SCS correction with Pearson correlation tests on the log2 fold change.

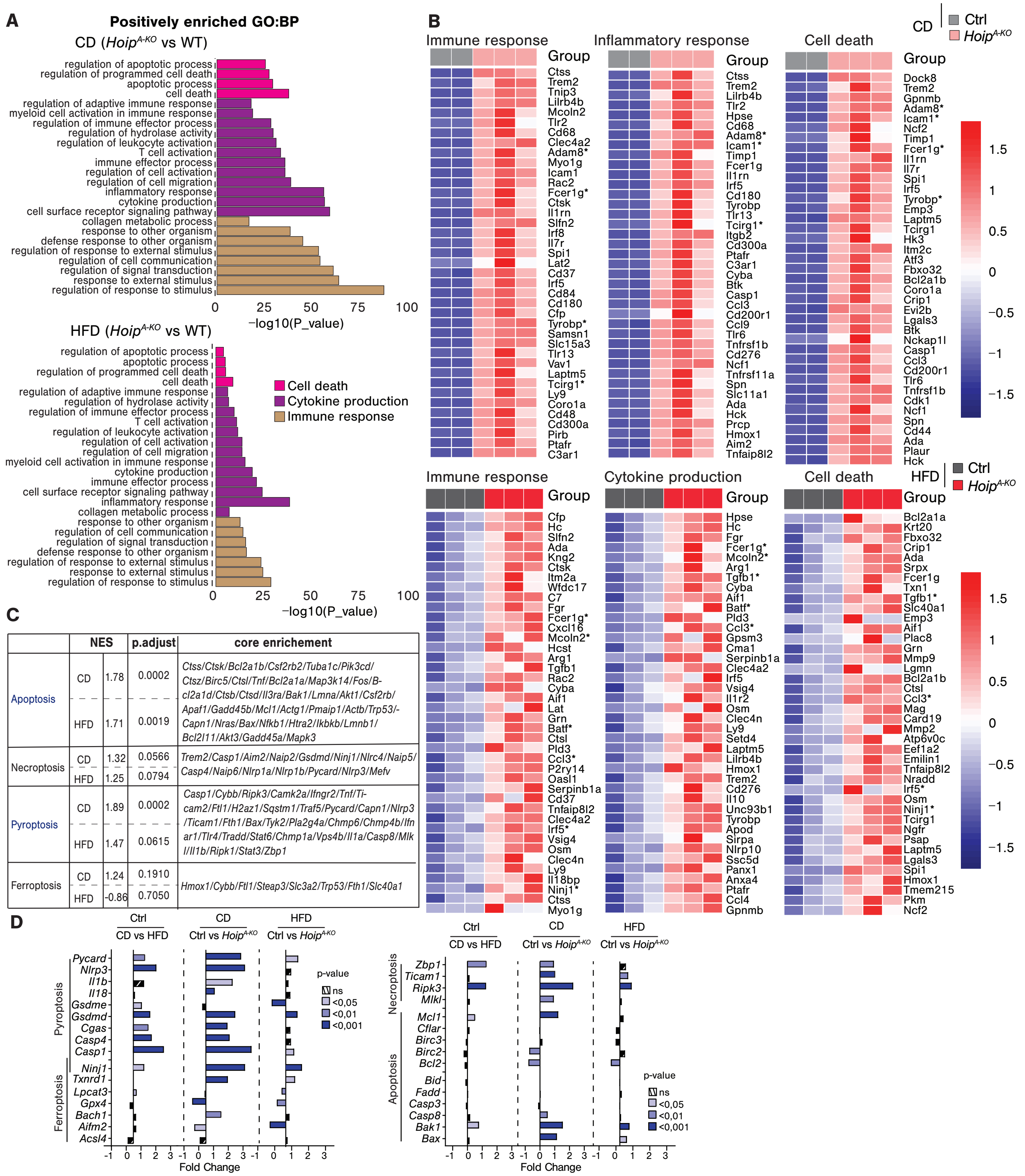

		NES	p.adjust	core enrichement
Apoptosis	CD	1.78	0.0002	Ctss/Ctsk/Bcl2a1b/Csf2rb2/Tuba1c/Pik3cd/Ctsz/Birc5/Ctsl/Tnf/Bcl2a1a/Map3k14/Fos/B-cl2a1d/Ctsb/Ctsd/Il3ra/Bak1/Lmna/Akt1/Csf2rb/Apaf1/Gadd45b/Mcl1/Actg1/Pmaip1/Actb/Trp53/-Capn1/Nras/Bax/Nfkb1/Htra2/Ikbkb/Lmnb1/Bcl2l11/Akt3/Gadd45a/Mapk3
	HFD	1.71	0.0019	
Necroptosis	CD	1.32	0.0566	Trem2/Casp1/Aim2/Naip2/Gsdmd/Ninj1/Nlrc4/Naip5/Casp4/Naip6/Nlrp1a/Nlrp1b/Pycard/Nlrp3/Mefv
	HFD	1.25	0.0794	
Pyroptosis	CD	1.89	0.0002	Casp1/Cybb/Ripk3/Camk2a/Ifngr2/Tnf/Ti-cam2/Ftl1/H2az1/Sqstm1/Traf5/Pycard/Capn1/Nlrp3/Ticam1/Fth1/Bax/Tyk2/Pla2g4a/Chmp6/Chmp4b/Ifnar1/Tlr4/Tradd/Stat6/Chmp1a/Vps4b/Il1a/Casp8/Mlkl/Il1b/Ripk1/Stat3/Zbp1
	HFD	1.47	0.0615	
Ferroptosis	CD	1.24	0.1910	Hmox1/Cybb/Ftl1/Steap3/Slc3a2/Trp53/Fth1/Slc40a1
	HFD	-0.86	0.7050	

Supplementary Figure 7: HOIP loss leads to an upregulation of genes involved in cell death and inflammation. Related to Figure 4 (A–D) Bulk RNA-seq analysis of GWAT after 16 weeks of diet (n:2–3 pools of 3 mice). (A) Positively enriched GO terms in CD HoipA-KO vs WT (left) and HFD *Hoip*$^{A\text{-}KO}$ vs WT (right). (B) Heatmaps of selected pathways from upregulated genes in *Hoip*$^{A\text{-}KO}$ mice. *indicates repeated genes. (C) Normalized enrichment scores (NES) and adjusted p-values (p.adjust) for selected cell death pathways (GSEA). (D) Shrunken fold change values of representative cell death pathway genes, normalized to controls. Statistics: For panels B and D, multiple comparisons in differential expression analysis (DEA) were corrected using the Benjamini-Hochberg method. Enriched pathways were identified using default parameters, including g_SCS correction and Pearson correlation tests on log2 fold change. GO terms are significant at adjusted p-value <0.05 (A). For panel C, GSEA uses permutation testing with p-values adjusted by the Benjamini-Hochberg procedure and reports q-values.

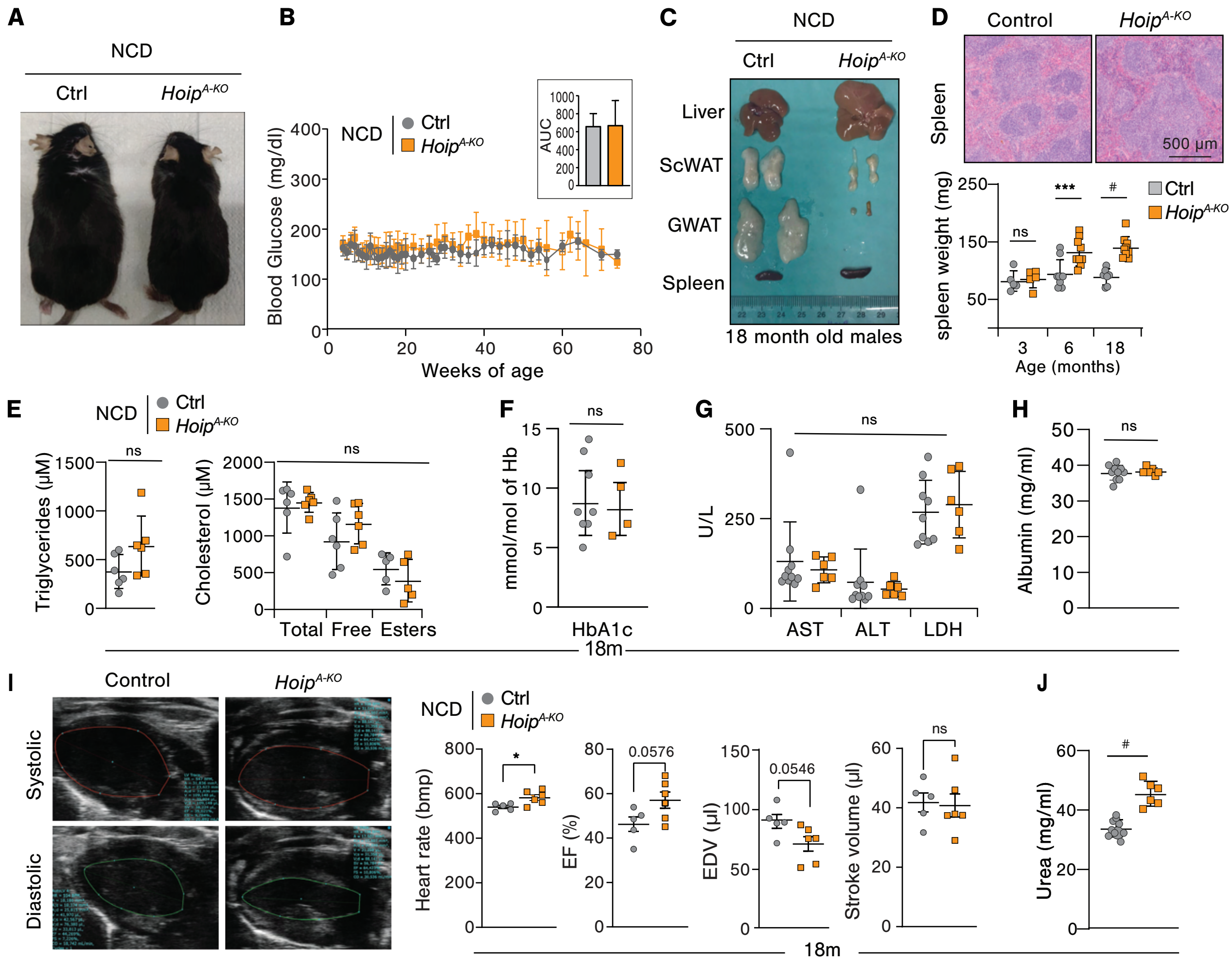

Supplementary Figure 8. *Hoip*$^{A-KO}$ lipodystrophic males do not develop dislipidemia during ageing. Related to Figure 4
(A) Representative image of 18-month-old mice under normal chow diet (NCD) at the endpoint. (B) Normal-fed Blood glucose monitoring under NCD (n: 14–16).(C) Representative images of the indicated tissues from 18-month-old mice. (D) Representative H&E images of spleens at 18 months of age (top) and spleen weight at 3, 6, and 18 months of age in male mice (bottom) (n: 5–10). (E–H) Serum levels of triglycerides and cholesterol (n: 5–6) (E), HbA1c (n: 4–8) (F), liver enzymes (AST, ALT, and LDH) (n: 6–10) (G), and albumin (n: 6–10) (H). (I) Representative echocardiographic images of mice at 18 months of age, and individual data for heart rate, ejection fraction (EF), end-diastolic volume (EDV), and stroke volume (n: 8–12; merged from two experimental cohorts). (J) Serum levels of urea (n: 6–10). All values represent mean ± SD. Statistics: For panels B, E, F, G, H, and J, t-test; for panel I, non-parametric Mann-Whitney test. p-values: () p<0.001, () p<0.01, () p<0.05, (ns) not significant.

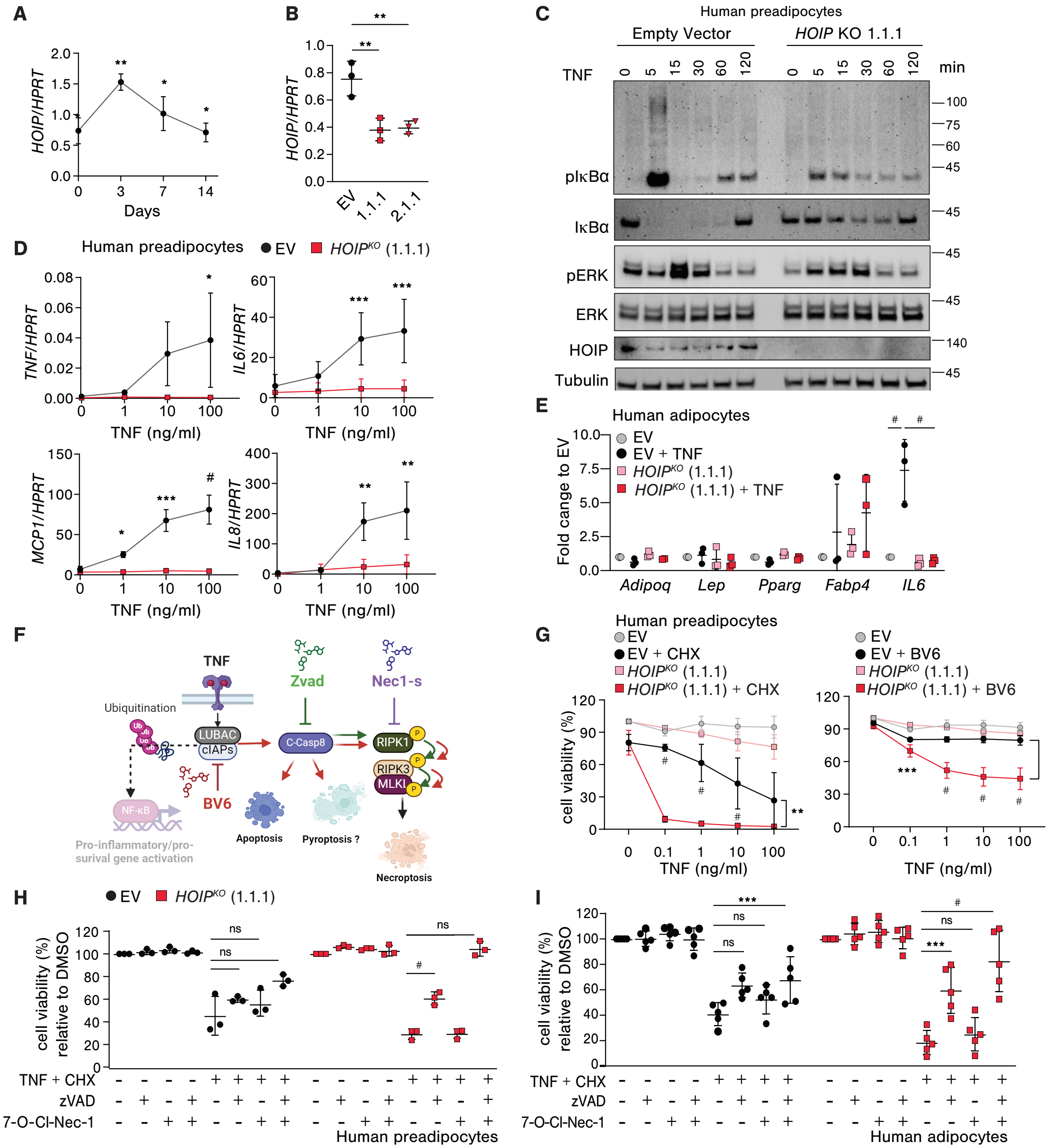

Supplementary Figure 9. HOIP is required for optimal NF-kB activation and protection from cell death upon TNF stimulation in human preadipocytes. Related to Figure 5

(A–B) HOIP expression levels normalized to HPRT in SGBS preadipocytes (PA) during adipogenic differentiation (A) and in engineered SGBS PA (B). Data represent three independent experiments performed in duplicates (n:3). EV: empty vector. (C) Western blot of the indicated proteins from human PA after treatment with 200 ng/ml TNF (n:2). (D) RT-qPCR analysis of the indicated genes normalized to HPRT in PA stimulated with increasing doses of TNF (n:3). (E) RT-qPCR analysis of adipogenesis genes in adipocytes in response to TNF (100 ng/ml). IL6 is included as a TNF response control; genes are normalized to untreated EV (n:3). (F) Diagram of components of the TNFR1 pathway targeted by the drugs used to study cell death. (G) SGBS PA viability after 24 h treatment with increasing doses of TNF in combination with cycloheximide (CHX, 50 µg/ml), Smac mimetic (BV6, 500 nM), or DMSO control (n:3). (H–I) Viability after 24 h treatment with 1 ng/ml TNF and 50 µg/ml CHX, either alone or combined with zVAD, Nec-1, or both, in PA (n:3) (H) or adipocytes (n:4) (I). All values represent mean ± SD. Statistics: Panel A: t-test; p-values: (**) p<0.01, (*) p<0.05, (ns) not significant. Panels B and E: one-way ANOVA for selected comparisons. Panels D, G, H, and I: two-way ANOVA with Bonferroni correction grouped by TNF concentration. p-values GP: (#) p<0.0001; () p<0.0002; (**) p<0.0021; (*) p<0.0332; (ns) p>0.0333.

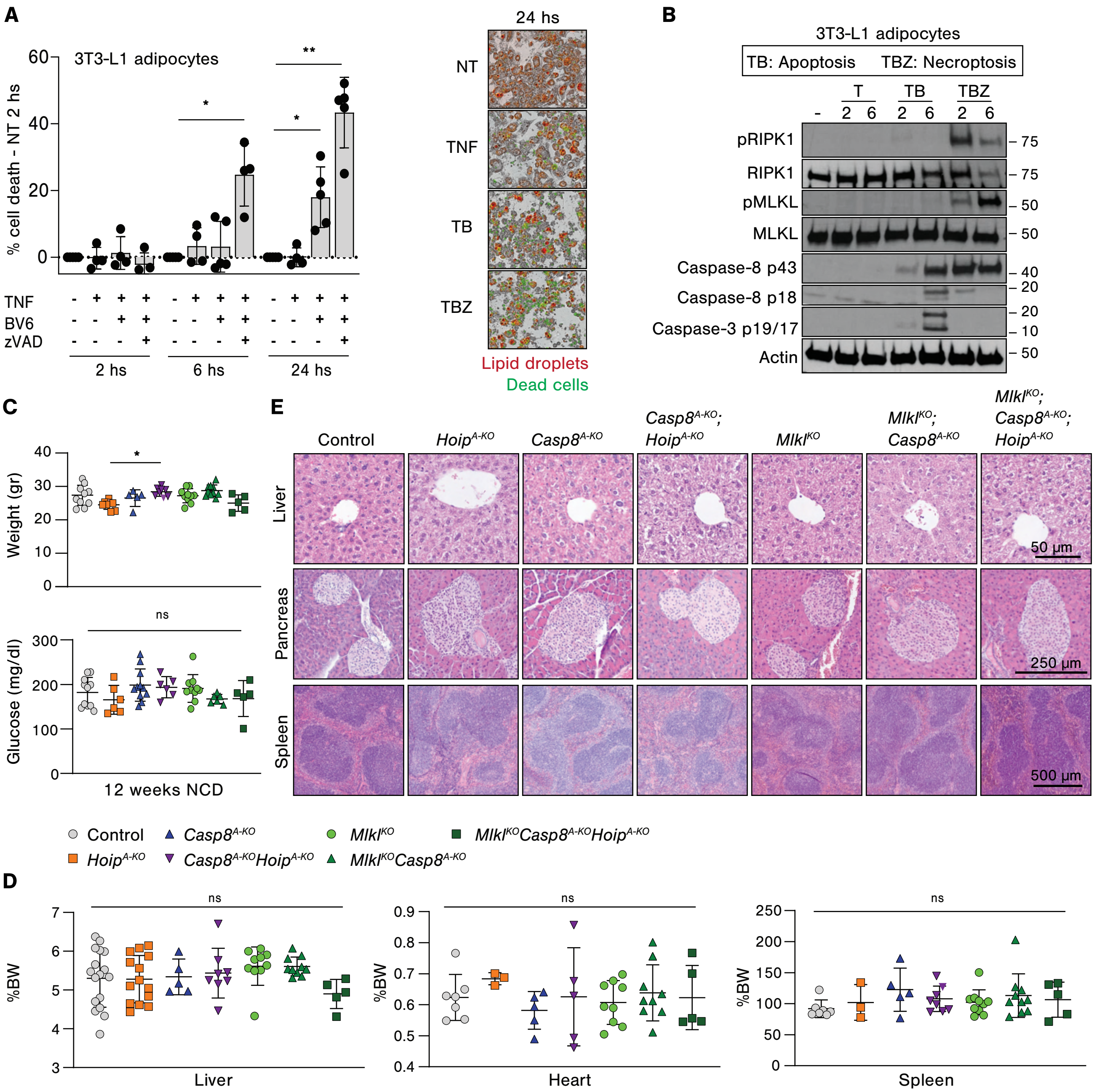

Supplementary Fig. 10. Caspase-8 deletion fully rescues *Hoip*$^{A-KO}$ spontaneous lipodystrophy. Related to Figure 6
(A) Percentage of cell death in 3T3-L1 differentiated mouse adipocytes treated with apoptotic (TNF+BV6) or necroptotic (TNF+BV6+zVAD) stimuli, measured by flow cytometry (left). The non-treated 2-hour condition was used as the basal level of cell death and subtracted from each independent experiment (n: 4). Representative images of mouse adipocytes after 24 hours of the indicated treatments are shown (right); green: dead cells, orange: lipid droplets. (B) Western blot for the indicated proteins from 3T3-L1 differentiated mouse adipocytes after the specified treatments (n: 4). (C–E) Evaluation of mutant 12-week-old males fed a normal chow diet (NCD) (n: 5–12): body weight and blood glucose at endpoint (C), tissue weight relative to total body weight (D), and representative H&E images of BAT and ScWAT (E). All values represent mean ± SD. Statistics: One-way ANOVA of selected groups with Bonferroni correction for multiple comparisons. p-values GP: (#) p<0,0001, (***), p<0,0002;(**) p<0,0021; (*) p<0,0332; (ns) p>0,0333.

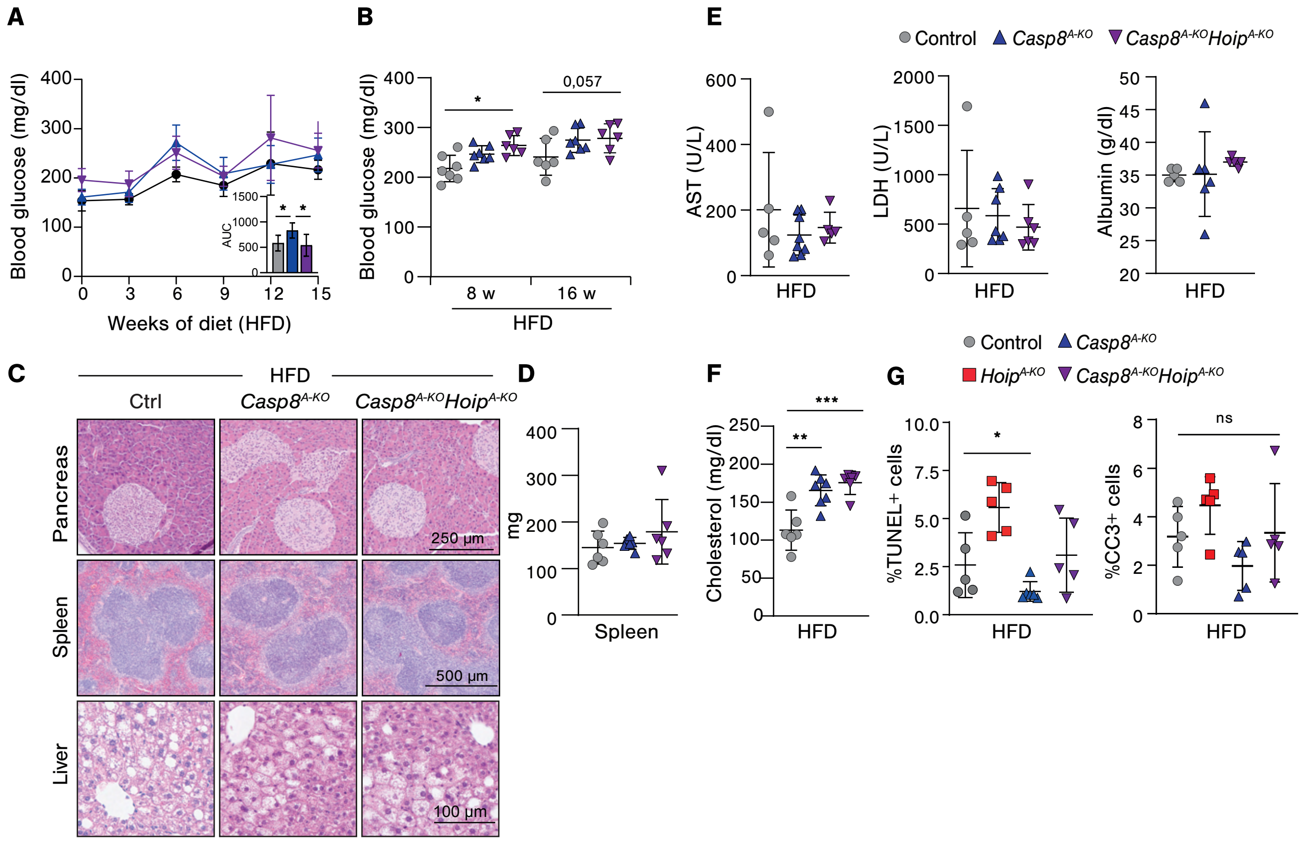

Supplementary Fig. 11. Caspase-8 deletion in adipocytes fully rescues *Hoip^A-KO^* predisposition to MASLD under HFD. Related to Figure 7

(A) Normal-fed blood glucose during HFD (n: 5–7). (B) Fasting glucose at 8 or 16 weeks of HFD (n: 5–7). (C) Representative H&E images of pancreas, spleen, and liver after 16 weeks of diet. (D) Spleen weights after 16 weeks of diet (n: 6–7). (E) Serum levels at 16 weeks of HFD: AST, ALT, and albumin and (F) total cholesterol (n: 5–7) (G) Quantification corresponding to Fig. 7I, expressed as the percentage of dead cells in GWAT (TUNEL or CC3+ cells) after 16 weeks of HFD (n: 5–6). All values represent mean ± SD. Statistics: One-way ANOVA with selected pair comparisons followed by Bonferroni correction. p-values GP: (#) p<0,0001, (***), p<0,0002;(**) p<0,0021; (*) p<0,0332; (ns) p>0,0333.

Supplementary Movies 1 and 2 legends:

Movie 1: GWAT live photon microscopy imaging for WT 12 weeks old males.

Representative video of 3 mice. 45 minutes before recording, the mice received bolus tail vein injections with the following markers: red: TMRE (mitochondrial membrane potential marker), blue: Hoechst (nuclear marker), pink: Propidium Iodide (cell death/membrane permeabilization), green: fluorescein-coupled dextran (blood flow). Epididymal fat was exposed and placed on a coverslip fixed on an image platform.

Movie 2: GWAT live photon microscopy imaging for *Hoip*$^{A\text{-}KO}$ 12 weeks old males.

Representative video of 3 mice. 45 minutes before recording, the mice received bolus tail vein injections with the following markers: red: TMRE (mitochondrial membrane potential marker), blue: Hoechst (nuclear marker), pink: Propidium Iodide (cell death/membrane permeabilization), green: fluorescein-coupled dextran (blood flow). Epididymal fat was exposed and placed on a coverslip fixed on an image platform.

Supplementary Tables S1-S5 are available as individual excel files containing:

Table S1: Lipidomic analysis for Triglycerides of livers after 16 weeks of Control Diet (CD) and 60% High fat diet (HFD).

Table S2: Lipidomic analysis for Diglycerides of livers after 16 weeks of CD and HFD.

Table S3: Lipidomic analysis for Cholesterol Esters of livers after 16 weeks of CD and HFD.

Table S4: Differential expression analysis (DEA) from Bulk RNAseq of GWAT after 16 weeks of CD and HFD.

Table S5: Data collection for correlation plots from Bulk RNAseq of GWAT after 16 weeks of CD and HFD.
